# Sour Tamarind Is More Antihypertensive than the Sweeter One, as Evidenced by In Vivo Biochemical Indexes, Ligand–Protein Interactions, Multitarget Interactions, and Molecular Dynamic Simulation

**DOI:** 10.3390/nu15153402

**Published:** 2023-07-31

**Authors:** Taslima Akter, Md. Rakibul Hassan Bulbul, Imran Sama-ae, M. A. Azadi, Kamrun Nahar Nira, Salahuddin Quader Al-Araby, Jobaier Ibne Deen, Md. Khalid Juhani Rafi, Srabonti Saha, Md. Muzahid Ahmed Ezaj, Md. Atiar Rahman

**Affiliations:** 1 Department of Biochemistry and Molecular Biology, University of Chittagong, Chittagong 4331, Bangladesh; taslimaakt22@gmail.com (T.A.); knnira@gmail.com (K.N.N.); sqarabi360@gmail.com (S.Q.A.-A.); jobaier.bmb@gmail.com (J.I.D.); krafi.cu@gmail.com (M.K.J.R.); srabonti7@cu.ac.bd (S.S.); 2 Institute for Developing Science and Health Initiatives, ideSHi, Dhaka 1216, Bangladesh; rakib@ideshi.org; 3 School of Allied Health Sciences, Walailak University, Nakhon Si Thammarat 80160, Thailand; imran.sa@wu.ac.th; 4 Department of Zoology, University of Chittagong, Chittagong 4331, Bangladesh; maazadi@yahoo.com; 5 Department of Genetic Engineering and Biotechnology, University of Chittagong, Chittagong 4331, Bangladesh; muzahid.geb@std.cu.ac.bd

**Keywords:** antihypertensive, *Tamarindus indica*, gamma-sitosterol, troponin I, dynamic simulation, network pharmacology

## Abstract

This research investigated the antihypertensive effects of tamarind products and compared their potentials based on an animal model’s data verified by molecular docking, multitarget interactions, and dynamic simulation assays. GC-MS-characterized tamarind products were administered to cholesterol-induced hypertensive albino rat models. The two-week-intervened animals were dissected to collect their serum and organs and respectively subjected to analyses of their hypertension-linked markers and tissue architectures. The lead biometabolites of tamarinds interacted with eight target receptors in the molecular docking and dynamic simulation studies and with multitarget in the network pharmacological analyses. The results show that the serum alanine aminotransferase (ALT), aspartate aminotransferase (AST), alkaline phosphatase (ALP), C-reactive protein (CRP), troponin I, and lipid profiles were maximally reinstated by the phenolic-enriched ripened sour tamarind extract compared to the sweet one, but the seed extracts had a smaller influence. Among the tamarind’s biometabolites, ϒ-sitosterol was found to be the best ligand to interact with the guanylate cyclase receptor, displaying the best drug-likeliness with the highest binding energy, −9.3 Kcal. A multitargeted interaction-based degree algorithm and a phylogenetic tree of pathways showed that the *NR3C1*, *REN*, *PPARG*, and *CYP11B1* hub genes were consistently modulated by ϒ-sitosterol to reduce hypertension and related risk factors. The dynamic simulation study showed that the P-RMSD values of ϒ-sitosterol–guanylate cyclase were stable between 75.00 and 100.00 ns at the binding pocket. The findings demonstrate that ripened sour tamarind extract may be a prospective antihypertensive nutraceutical or supplement target affirmed through advanced preclinical and clinical studies.

## 1. Introduction

Hypertension is a major risk factor for cardiac disease and stroke, with an increase in risk for these ailments with progressively higher blood pressure, dyslipidemia, and obesity. The total lipid profile of an individual is a contributive factor resulting from their blood cholesterol and its associated varieties of lipoproteins, i.e., high-density lipoproteins (HDL, or α-lipoproteins), low-density lipoproteins (LDL, or β-lipoproteins), very-low-density lipoproteins (VLDL, or pre-β-lipoproteins), and triglycerides. The blood pressure level and coronary heart diseases have a strong correlation with the lipid profile, particularly with the blood cholesterol level [1].

The interrelationship between liver dysfunction and the development of hypertension is being increasingly recognized. The liver is a vital organ in metabolism and plays numerous roles, including the synthesis, degradation, storage, and biotransformation of biomolecules in the human body [2]. The liver enzymes alanine and aspartate aminotransferase (ALT and AST), γ-glutamyltransferase (GGT), and alkaline phosphatase (ALP) have been widely used as good markers of liver health, and some epidemiological studies have demonstrated an association of ALT and GGT with metabolic syndrome, CVD, and type 2 diabetes [3].

The contraction of smooth muscle cells is thought to be related to a rise in intracellular calcium concentration, which may explain the vasodilatory effect of calcium channel-blocking drugs. Prolonged contraction of SMC is thought to induce structural changes with the thickening of the arteriolar vessel walls, possibly mediated by angiotensin, and leading to an irreversible rise in peripheral resistance. Cardiac output is related to sympathetic overactivity [4].

Phytochemicals are mainly classified as flavonols, flavones, flavanones, isoflavones, catechins, anthocyanidins, or chalcones. The common feature of flavonoid compounds is their phenyl benzopyrone skeleton (C6-C3-C6) [5,6], and they are antioxidants that often function as reducing agents, such as thiols, ascorbic acid, or polyphenols. They exist as vitamins, minerals, and other compounds in foods [6]. The antioxidant property is mainly brought about by the presence of polyphenolic compounds, such as anthocyanins, flavonoids, phenolic acids, and phenolic diterpenes. *T. indica* L. is said to contain a large number of polyphenolic compounds with the potential for antioxidant activity [7].

Modern medical science has developed several synthetic drugs and therapeutics with improved efficacy to treat cardiovascular diseases, including hypertension, but they possess a significant number of side effects. Herbal medicines, therefore, have been regaining importance because of their ease of availability, fewer side effects, and cost-effectiveness [8]. Ethnobotanical surveys of various medicinal plants indicate their vast use in the treatment of cardiovascular disorders. For example, plants such as *Syzyium glineens*, *Tamarindus indica*, *Passiflora nepalensis* wall, etc. have been used for the treatment of hypertension [9].

*Tamarindus indica,* a Fabaceae family member, also referred to as tamarind, is frequently used in traditional cooking and Ayurvedic herbal remedies. According to numerous studies, *T. indica* is a multifunctional tree, with every part of the tree having at least some nutritional or therapeutic benefit. Proanthocyanidins, which are oligomeric flavonoids also known as condensed tannins, are used as powerful antioxidants and are usually present in the peels of fruits and vegetables. They are among the phenolic antioxidants present in the tamarind pericarp [10]. In different parts of Bangladesh, there are various varieties of tamarind, especially sour and sweet types, but the full extent of their health benefits has not yet been discovered. Akter et al. (2022) recently reported the cardioprotective role of sour tamarind, while the sweeter one was unaddressed [11]. Considering the foregoing, the current investigation was undertaken to assess the antihypertensive effects of different types of *T. indica* extracts on cholesterol-induced hypertensive Wistar albino rats. The results were further verified by a comprehensive computational biological study using target ligand–receptor interactions, and network pharmacological and biological simulation assays.

## 2. Materials and Methods

### 2.1. Chemicals and Reagents

All reagents used in this investigation were of analytical grade unless explicitly described otherwise. Cholesterol (CAS No: 57-88-5) was purchased from Sigma-Aldrich (St. Louis, MO, USA), Avastrol (Atorvastatin) (Albion Laboratories Ltd., South Rahmatnagar, Barabkunda, Sitakunda, Chittagong, Bangladesh), methanol (CAS No: 67-56-1), hydrochloric acid (CAS No: 7647-01-0), sodium hydroxide (CAS No: 1310-73-2), aluminum chloride (CAS No: 7446-70-0), sodium carbonate (CAS No: 497-19-8), potassium ferricyanide (III) (CAS No: 13746-66-2), iodine (CAS No: 7553-56-2), and potassium iodide (CAS No: 7681-11-0) were purchased from Sigma-Aldrich (St. Louis, MO, USA). Folin–Ciocalteu reagent, gallic acid (CAS No: 149-91-7), vanillic acid (CAS No: 121-34-6), rutin (CAS No: 153-18-4), and catechin (CAS No: 154-23-4) were also purchased from Sigma-Aldrich (St. Louis, MO, USA).

### 2.2. Collection of Sample Material

Sour and sweet *Tamarindus indica* fruits were used as samples in this investigation. Sour *T. indica* fruits were collected from the local market of Chittagong University (GPS: 22.479827, 91.793494), and sweet varieties of *T. indica* were collected from the busiest local market of Cox’s Bazar (GPS:21°25′38″ N 92°00′18″ E). The samples were authenticated as *T. indica* by Dr. Shaikh Bokhtear Uddin, Taxonomist, and Professor, Department of Botany, University of Chittagong. Voucher specimens of the samples were preserved under their accession numbers (sour variety-CAMH19/03, sweet variety-BCAMH19/04).

### 2.3. Preparation of Extract from the Flesh of Tamarind

Immediately after the collection of *T. indica*, the skin of the fruits was removed, and the fleshy pericarp and hard seeds were separated from each other. The tamarind flesh was then mixed with water and pulverized by a mechanical blender machine (Sonipat, Heavy Duty Electric Dry Masala & Herbs Grinder, Swing Type 2300 W Haryana, India) to make a juicy extract of the tamarind. The seeds of ripened sour tamarind were dried and ground with an iron mortar and pestle. The crushed seeds were then mixed with water to make an aqueous extract of the seeds. All of the extracts were filtered by cheese clothes and then kept in an incubator at a 45 °C temperature. The crude materials of the different semisolid extracts of the tamarind parts are henceforth known as the flesh of ripened sour tamarind (FRiST), the seeds of ripened sour tamarind (SRiST), the flesh of raw sour tamarind (FRaST), and the flesh of sweet tamarind (FSwT). The samples were preserved in Eppendorf tubes covered with parafilm, and sieves were put on the caps of the tubes. After 3 days of refrigeration at −80 °C, the samples were lyophilized by a Labconco freeze drier (Labconco 7934000, Kansas City, MO, USA). The lyophilized samples were stored at −40 °C for future use.

### 2.4. Standardization of the Extraction Procedure

An established method was used to optimize the crude extraction process [12]. The dried materials were quickly extracted using water at 23 ± 0.5 °C. A central composition design was used to assess the impact of the two factors: the extraction time (T: 36–72 h) and the solvent–sample ratio (SSR: 2/1 to 4/1), represented as the milliliters of solvent per gram of dried material. The solvent was evaporated from the extract using a vacuum evaporator (RE 200, Bibby Sterilin Ltd., Staffordshire, UK) and filter paper (Whatman #1).

### 2.5. Screening for the Phytochemical Content of Prepared Aqueous Extracts

The total flavonoid content (TFC), total phenolic content (TPC), total proanthocyanidin content (TPrAC), and total antioxidant capacity (TAC) of the FRiST, SRiST, FRaST, and FSwT were determined.

The total flavonoid contents (TFCs) of the aqueous extracts were determined according to the method established by Kumaran and Karunakaran et al. (2007) [9]. The total phenolic contents (TPCs) of the four samples of tamarinds were measured according to a method described by Singleton and Rossi et al. (1965) [13]. Quantitative estimations of the proanthocyanidins (TPrAC) and total antioxidant (TA) capacity were carried out using the modified HCl–vanillin method established by Abdelseed et al. (2011) [14].

### 2.6. GC-MS Analysis of FRiST and FSwT

The crude materials of the 1FRiST and FSwT were analyzed by GC-MS using electron impact ionization (EI) with a gas chromatograph (GC-17A, Shimadzu Corporation, Kyoto, Japan) coupled to a mass spectrometer (GC-MS TQ 8040, Shimadzu Corporation, Kyoto, Japan). A fused silica capillary column (Rxi-5ms; 0.25 m film thickness) was coated with DB-1 (J&W). The inlet temperature of the capillary was set at 260 °C and the oven temperature was set at 70 °C (0 min), and then 10 °C and 150 °C (5 min); 12 °C and 200 °C (15 min); and 12 °C and 220 °C (5 min), respectively. The column flow rate was 0.6 mL/min helium gas at a constant pressure of 90 KPa. The auxiliary (GC to MS interface) temperature was set at 280 °C. The MS was set to scan mode with a scanning range of 40–350 amu. The mass range was set in the range of 50–550 *m*/*z*. The prepared sample was then run for GC/MS analysis. The total GC-MS running time was 35 min. All peak areas were compared with the database in the GC-MS library version NIST 08-S.

### 2.7. Qualitative Identification of Carbohydrates, Proteins, Alkaloids, Glycosides, Tannins, Saponins, Phenolic Compounds, Steroids, Triterpenoids, and Flavonoids

The presence of carbohydrates was determined by Fehling’s test, Fehling’s A, and Fehling’s B reagent. The presence of protein, alkaloid, glycoside, tannins, saponins, phenolic compounds, steroids, and triterpenoids was tested by the Biuret test, Wagner’s test, the glacial acetic acid test, the lead acetate test, the saponins test, Salkowski’s test, and the alkaline reagent test, respectively [15,16].

### 2.8. Experimental Animals and Their Maintenance

The antihypertensive activity of the extract was carried out on 38 male Wistar albino rats aged 6–8 weeks (average weight, 150–200 g) before the experiment. The animals were housed in standard environmental conditions under a 12/12 h light/dark natural cycle in the animal house of the Department of Biochemistry and Molecular Biology, University of Chittagong. The animals were individually housed in a polycarbonate cage bedded with wood husk at a temperature of 22 ± 2 °C and humidity of 55–60%. All animals had free access a to standard diet and tap water. The ARRIVE ethical guidelines, based on approval from the Ethical Review Board of the Faculty of Biological Sciences, University of Chittagong, verified the animal care, dosing, and sacrifice (AERB_FBSCU_20230213(6)).

### 2.9. Induction of Hypertension Using Cholesterol

The experimental Wistar albino rats (Age: 7–8 weeks, body weight: 180–200 g) of the assigned rats were randomly divided into control and treatment groups comprising 4 animals each. The normal control group (non-hypertensive) received vehicles only. The hypertensive control group developed hypertension but was left untreated. The reference control group developed hypertension and was treated with a reference drug, atorvastatin. The treatment groups were administered two different doses (50 mg and 100 mg/kg BW) of the FRiST, SRiST, FRaST, and FSwT aqueous extracts. All groups except the negative control groups were fed cholesterol (150 mg/kg BW) dissolved in vegetable oil to induce hypertension. Meanwhile, the eight treatment groups were treated with aqueous extracts of FRiST, SRiST, FRaST, and FSwT, at doses of 50 mg/kg and 10 mg/kg BW each. The treatment continued for 14 days.

#### 2.9.1. Recording the Animals’ Body Weights and Collection of Blood and Organs

The body weights of the intervened animals were recorded each week, and the treatments were provided by feeding needle. The animals were sacrificed after two weeks of intervention, and their blood, liver, and hearts were taken in heparinized test tubes. The blood samples were centrifuged at 3000 rpm for 15 min at 20 °C to separate the serum, which was further analyzed for biochemical analyses. The liver and heart were collected from each animal and washed with 0.9% NaCl (normal saline). The organs were then wiped with tissue paper and weighed. The weighed organs were preserved with 4% buffered formalin within the plastic vials. The weights of the liver and heart were recorded. The liver was used to determine the glycogen level. The hearts were used for histopathological investigation. The liver glycogen concentrations were measured by a phenol–sulfuric acid method as described by [15].

#### 2.9.2. Biochemical Analyses of Serum

Serum biochemical parameters, including the aspartate aminotransferase (AST), alanine aminotransferase (ALT), alkaline phosphatase (ALP), lipid profile, troponin I, C-reactive protein (CRP), creatinine kinase (CK-MB), lactate dehydrogenase (LDH), and creatinine levels, were measured using reaction kits on a semi-autoanalyzer (Humalyzer 3000, Human).

#### 2.9.3. Liver Glycogen Estimation

The determination of hepatic glycogen was performed by the phenol–sulfuric acid method as described by Lo et al., (1970) [15]. The standard-1 solution, which was generated at a concentration of 1000 g/mL, was diluted four more times to create the standard-2 (500 g/mL), standard-3 (250 g/mL), standard-4 (125 g/mL), and standard-5 (62.5 g/mL) solutions. These were the standard solutions for this experiment. The liver samples (about 80 mg) were transferred to test tubes containing 30% KOH (*w*/*v*), which was saturated with Na_2_SO_4._ The liver tissues dissolved in KOH were then boiled for 30 min until complete homogenization occurred. The homogenized mixture was cooled in ice. The glycogen was precipitated by adding 2 mL 95% ethanol and then incubating the mixture on ice for 30 min. The test tubes were then centrifuged at 840× *g* (3000 rpm) for 20–30 min. The supernatants were discarded, the precipitate was dissolved in 3 mL of distilled water, and an aliquot was then diluted 1:4 (a 100 µL aliquot in 400 µL of distilled water). The standard was also started from here. Both the sample and the standards were run in duplicate. First, 1 mL of 5% phenol and then of 5 mL 96% H_2_SO_4_ was added to the solution, which was left to stand for 10 min. The solutions were incubated at 25–30 °C for 15 min. The absorbances (OD) of the solutions were measured at 490 nm. The glycogen concentrations of the tissues were calculated using the following equation [15].

Liver glycogen (mg/g tissue) = A/k × V/v × 10^−4^/w

where

k = the slope of the standard curve;

V = the total volume (mL) of the glycogen solution;

v = the volume (mL) of the aliquot to which phenol–sulfuric acid solution is added;

A = absorbance at 490 nm;

w = sample weight (g).

#### 2.9.4. Histopathological Analyses

Immediately after sacrifice, the tissues were collected and preserved in 4% buffered formalin for 48 h. The formalin was changed every week until the histopathological analysis was performed. Vertical sections of the tissues were taken by sharp blade for further processing. The tissues were dehydrated by passing them through ascending grades of ethanol (70%, 80%, 90%, and 100% *v*/*v*) for one hour in each solution. The tissues were passed through 100% ethanol three times, for a total of 3 h. The samples were transferred through progressively more concentrated ethanol to remove the water from the tissue. To remove alcohol from the tissues, they were passed through a xylene solution three times consecutively for of total three hours, one hour per xylene solution. The tissues were then embedded in molds along with molten paraffin wax, and then were allowed to cool and harden, and preserved at 4 °C until cross-section was done. The embedded tissues were sectioned at 5 μm using a semi-automated rotator microtome (Biobase Bk-2258, Laboratory Manual Microtome, Jinan, China). The tissue sections were then mounted on glass slides using an incubator at 60–70 °C for 30 min. Afterward, the tissue sections were deparaffinized with xylene and rehydrated by using different graded ethanol dilutions (100%, 90%, and 70%). The sections were stained with hematoxylin and eosin (H & E). All slides of the liver and heart were examined under an Olympus BX51 Microscope (Olympus Corporation, Tokyo, Japan), and the histopathological images were taken with the help of the Olympus DP20 system under a magnification of X200.

### 2.10. Statistical Analysis

All data are presented as the mean ± standard error of the mean. The data were analyzed by Statistical Package for Social Sciences (SPSS, Version 22.0, IBM Corporation, New York, NY, USA) using one-way analysis of variance (ANOVA), followed by Tukey’s post hoc tests for multiple comparisons. The values were considered significantly different at *p* < 0.05.

### 2.11. In Silico Approaches

#### 2.11.1. Molecular Docking Analysis

Based on a review of the literature, the receptors/enzymes were chosen for the analysis of molecular interactions to reveal anti-hypertensive activity [16,17,18]. The 3D crystal structures of tyrosine hydroxylase (PDB ID: 1TOH), the BETA-1 subunit of the soluble guanylyl cyclase (PDB ID: 3HLS), human high-conductance Ca^2+^-gated K^+^ channel (BK channel) (PDB ID: 3NAF), nuclear hormone receptor PPAR-gamma (PDB ID: 3R8 A), human angiotensin receptor (PDB ID: 4Y AY), macrocyclic IL-17A antagonists (PDB ID: 5H I3), and human soluble guanylate cyclase (PDB ID: 6JT0) were imported from the RCSB Protein Data Bank (PDB), an online database (https://www.rcsb.org/, (accessed on 22 December 2022)). The Discovery Studio protein preparation protocol was used to accomplish tasks, such as inserting missing atoms into incomplete residues and removing water molecules and ligands from proteins. Before processing, hydrogen atoms were also introduced to the protein molecules. The best binding sites were selected by using the online tool PockDrug [19]. The PubChem repository (https://pubchem.ncbi.nlm.nih.gov/, (accessed on 22 December 2022)) was used to extract the chemical structure of the main identified compounds from *T. indica* using GC-MS. The molecular docking research followed Hossen et al.’s methodology and was briefly described in [20].

#### 2.11.2. Evaluation of Pharmacokinetic Parameters

Lipinski’s rule of fives [21] and Veber’s rules (number of rotatable bonds; topological polar surface area) were used to assess the absorption, distribution, metabolism, excretion, and toxicity (ADME/T) characteristics of bioactive compounds from AOME [22]. QikProp (Schrödinger Release 2017-1: QikProp, Schrödinger, LLC, New York, NY, USA) and SwissADME (http://www.swissadme.ch/, (accessed on 22 December 2022)) were used to evaluate the ADME/T properties. QikProp and SwissADME are powerful ADME/T prediction tools that predict whether a compound’s ADME/T performance will be acceptable.

#### 2.11.3. Determination of Toxicological Properties

The AdmetSAR online tool was used to establish the toxicological properties of the selected compounds, as toxicity is a major worry during the development of novel drugs [23]. The Ames toxicity, carcinogenic properties, acute oral toxicity, and acute rat toxicity were all predicted in this research.

### 2.12. Building the Bioactive Compound–Target Protein Network

Target proteins associated with bioactive phytochemicals were discovered using SwissTargetPrediction (http://www.swisstargetprediction.ch/, (accessed on 22 December 2022)), which is based on network pharmacology-based prediction. Each protein–chemical interaction received a score. To match their possible targets, bioactive compounds (eight for GC-MF and all compounds for LC-MS-MS) were entered into SwissTargetPrediction collectively. The organism chosen was “Homo sapiens”, and the median necessary interaction score was set at 0.4. We also utilized GeneCard (https://www.genecards.org, (accessed on 22 December 2022)), which predicts the matching possible bioactive targets with a probability greater than 0.1 for hypertension. Further investigation did not consider compound targets with no connection to the interactions between the compounds and proteins. The target genes of the Swiss TargetPrediction and GeneCard were compared using the online application “Calculate and build custom Venn diagrams” (http://bioinformatics.psb.ugent.be/webtools/Venn/, (accessed on 22 December 2022)).

### 2.13. Development of the Anticipated Genes’ Protein–Protein Interaction (PPI) Networks

By using the Search Tool for the Retrieval of Interacting Genes (STRING) database (https://string-db.org/cgi/input.pl, (accessed on 22 December 2022); STRING-DB v11.0), we created a PPI network of the predicted genes. Using the Cytoscape plugin cytoHubba, the rank of the target proteins was determined based on the strength of connections in the PPI network. To create a PPI protein interaction network, the collected protein interaction data of the target proteins were imported into the Cytoscape 3.9.1 program.

### 2.14. Pathway Enrichment Analysis of the Target Proteins Using Gene Ontology (GO) and the Kyoto Encyclopedia of Genes and Genomes (KEGG)

The Database for Annotation, Visualization, and Integrated Discovery (DAVID, https://david.ncifcrf.gov/, (accessed on 22 December 2022)) v6.8 was used to determine the function of target proteins that interact with the active components in gene function and signaling pathways. The projected genes were substantially related to the KEGG pathways, which were found. We examined the KEGG pathway enrichment and gene ontology (GO) function of identified genes. The target proteins connected to the KEGG pathways, cellular components (CCs), molecular functions (MFs), and biological processes (BPs) were also characterized. A *p*-value of 0.05 or below was regarded as significant.

### 2.15. Molecular Dynamics (MD) Simulations

MD simulations of the thermolysin–gamma-sitosterol complex utilized the Schrödinger suite’s Desmond module [24]. Hydrogen bonds were allocated using standard protocols throughout this process. The thermolysin–gamma-sitosterol complexes were then subjected to the optimal potentials for liquid simulations (OPLS) force field. After immersing the complexes in a transferable intermolecular potential with 3 points (TIP3P) water model at a distance of 10 from the center of the box, the energy of the complexes was reduced. After that, the system was neutralized by adding sodium and chloride ions to simulate an in vivo environment. Next, molecular dynamic simulations were run for 100 ns, with ensembles of constant particle numbers, pressure, and temperature (NPTs) with a recording interval of 100 ps [25]. The temperature and pressure were adjusted to 310.15 K and 1.01325 bar, respectively, to simulate the in vivo environment [26]. The simulation interaction diagram tool in the Schrödinger suite’s Desmond module was used to investigate the results [24].

## 3. Results

### 3.1. Phytochemical Screening

The identification tests for the carbohydrates, proteins (Biuret test), alkaloids, tannins (lead acetate test), saponins (saponins test), steroids, and triterpenoids (Salkowski’s test), polyphenols and flavonoids (alkaline reagent test) showed positive results, indicating the presence of these phytochemical constituents in the aqueous extracts of the pericarp of ripened sour *T. indica* (FRiST). On the other hand, the test for the glycoside bond showed a negative result for the aqueous extracts of FRiST. The results are shown in Table 1.

### 3.2. TFC, TPC, TPrAC, and TAC of FRaST, FRiST, FSwT, and SRiST Extracts

The TFC was estimated by the standard rutin curve (y = 0.002x + 0.174, R^2^ = 0.426)) and expressed as the rutin equivalents per gram of the plant extract. The TFCs of the four different samples were found to be 95.33 mg rutin/g for FRiST and FRaST, 115.5 mg rutin/g for FSwT, and 173.8 mg rutin/g for SRiST. Among the 4 samples, SRiST possessed the highest quantity of TFC. The TPCs, using the Folin–Ciocalteu reagent method, were found to be 0.15 mg GAE/µg for the FRiST sample, 0.24 mg GAE/µg for both the FRaST and FSwT, and 0.063 mg GAE/µg for the TB. The TPrACs of the four *T. indica* extracts were found to be 2.663 mg/g of dry weight for FRiST, 15.4 mg/g dry weight for FRaST, 4.55 mg/g dry weight for FSwT, and 8.44 mg/g dry weight for SRiST. The data show that water might not be a good extract for proanthocyanidin elucidation. The data are presented in Table 2.

### 3.3. Effect of the Tamarind Products on the Body and Organ Weights of Experimental Animals

**Figure 1:**
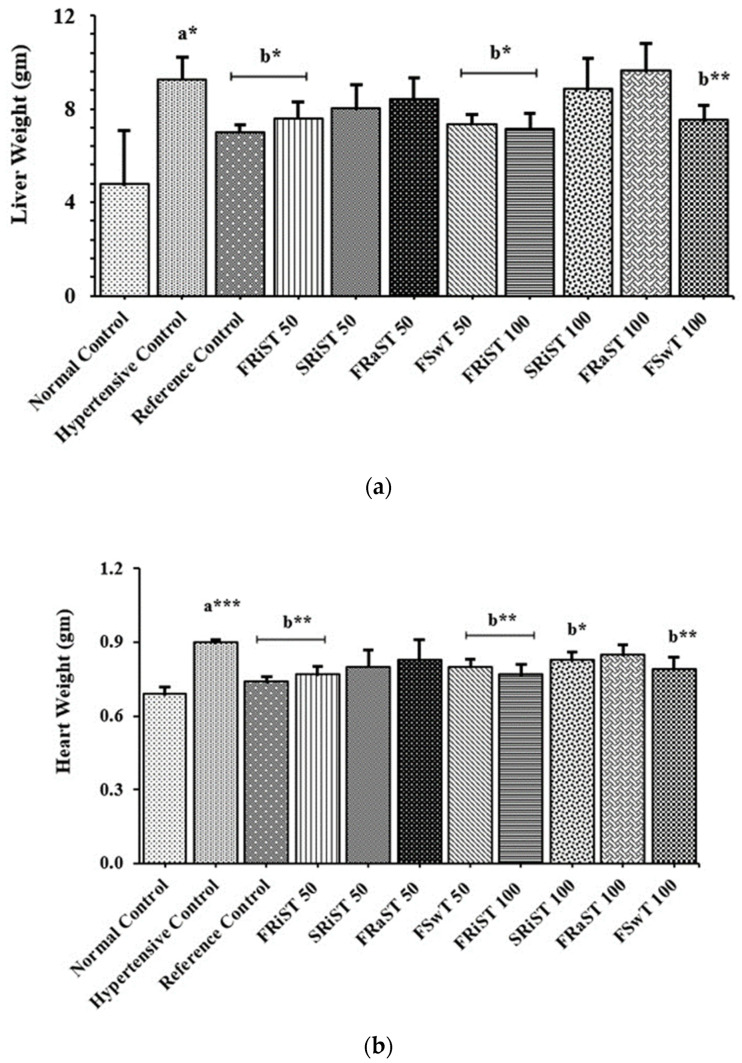
Effects of tamarind products on liver weight (**a**) and heart weight (**b**). Each bar represents the mean ± SD for *n* = 5 as analyzed by one-way ANOVA followed by *t*-tests. FRiST 50 and FRiST 100 = flesh of ripened sour tamarind aqueous extract at 50 mg/kg BW and 100 mg/kg BW; SRiST 50 and SRiST 100 = seeds of ripened sour tamarind aqueous extract at 50 mg/kg BW and 100 mg/kg BW; FRaST 50 and FRaST 100 = flesh of raw sour tamarind aqueous extract at 50 mg/kg BW and 100 mg/kg BW; FSwT 50 and FSwT 100 = flesh of sweet tamarind aqueous extract at 50 mg/kg BW and 100 mg/kg BW. Normal control vs. hypertensive control: *p* < 0.05 = a*; *p* < 0.001 = a***; hypertensive control vs. treatment controls: *p* < 0.05 = b*; *p* < 0.01 = b**; Normal control group = non-hypertensive animals; hypertensive control group = developed hypertension and left untreated; reference control group = developed hypertension and treated with a reference drug.

The positive control (PC, atenolol) and all of the treatment groups except SRiST100 were observed to inhibit weight gain in the animals, while the normal control groups showed a significant (*p* < 0.05) increase in body weight (Table 3). Cholesterol was found to induce the rats to gain weight, whereas the reference control and the treatments with FRiST50, FRiST100, FRaST50, FRaST100, and FSwT100 were shown to effectively inhibit the gain of animal body weight. However, the weight gain of the SRiST100 group was insignificant in comparison to the NC groups. The sour and sweet tamarinds equally contributed to protecting the liver and heart weights of the experimental animals. The FSwT50 and 100 and FRiST50 and 100 treatments were recognized to significantly improve the liver weight compared to the positive control, while the same doses were observed to reduce the heart weight significantly, implying their consistent effect in an antihypertensive role. Figure 1 displays the comparative relative weights of the livers and hearts of the intervened animals.

### 3.4. Effects of Tamarind Products on Biochemical Parameters

#### 3.4.1. Effects of the Extracts on Serum Lipid Profile

The effects of tamarind products on the serum lipid profile, including the total cholesterol (TC), triglycerides (TGs), LDL, VLDL, and HDL, are summarized in Table 4. The flesh of ripened sour tamarind (FRiST50) at 50 mg/kg body was found to maximally reduce the total cholesterol level. All the treatment groups except sweet tamarind (FSwT50) and raw sour tamarind (FRaST100) significantly (*p* < 0.05) minimized the cholesterol levels compared to the normal control group. The flesh of raw sour tamarind (FRaST50) achieved the highest reduction of TG levels, while SRiST50, FSwT, and FSwT were also found to significantly minimize the TG levels compared to the positive control. All of the treatments significantly (*p* < 0.01) downturned the LDL levels and increased the HDL levels compared to the hypertensive control group. Lower doses of all the treatments displayed a VLDL decrement, which was statistically significant compared to the hypertensive group. Interestingly, FSwT100 showed no VLDL-reducing effect. 

#### 3.4.2. Effect of the Extracts on Serum Enzyme Activities

The changes in the ALT, AST, and ALP levels at the end of the intervention are presented in Table 5. The serum ALT level was found to be maximally attenuated by the FRiST50 and FSwT100, while the other treatments also showed a significant reduction of the ALT levels. The FRiST50 and FSwT100 treatments, among all other groups, showed the highest decrements in the AST levels, and the values were statistically significant (*p* < 0.05) compared to both the hypertensive group and positive control (PC) group. Excitingly, the SRiST50 was found to show the maximum significant decrease in the ALP levels; while it was very consistent in reducing the ALT, AST, and ALP levels.

#### 3.4.3. Effects of the Extracts on the CRP, Troponin I (cTnI), and Liver Glycogen Levels

**Figure 2:**
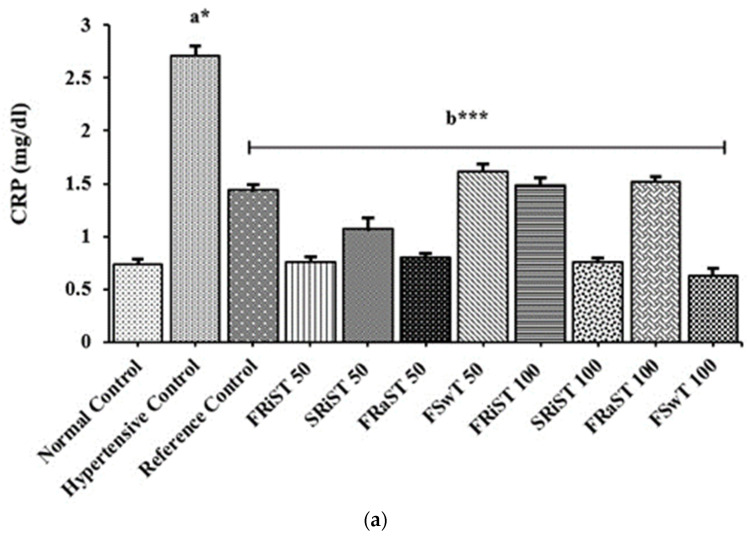

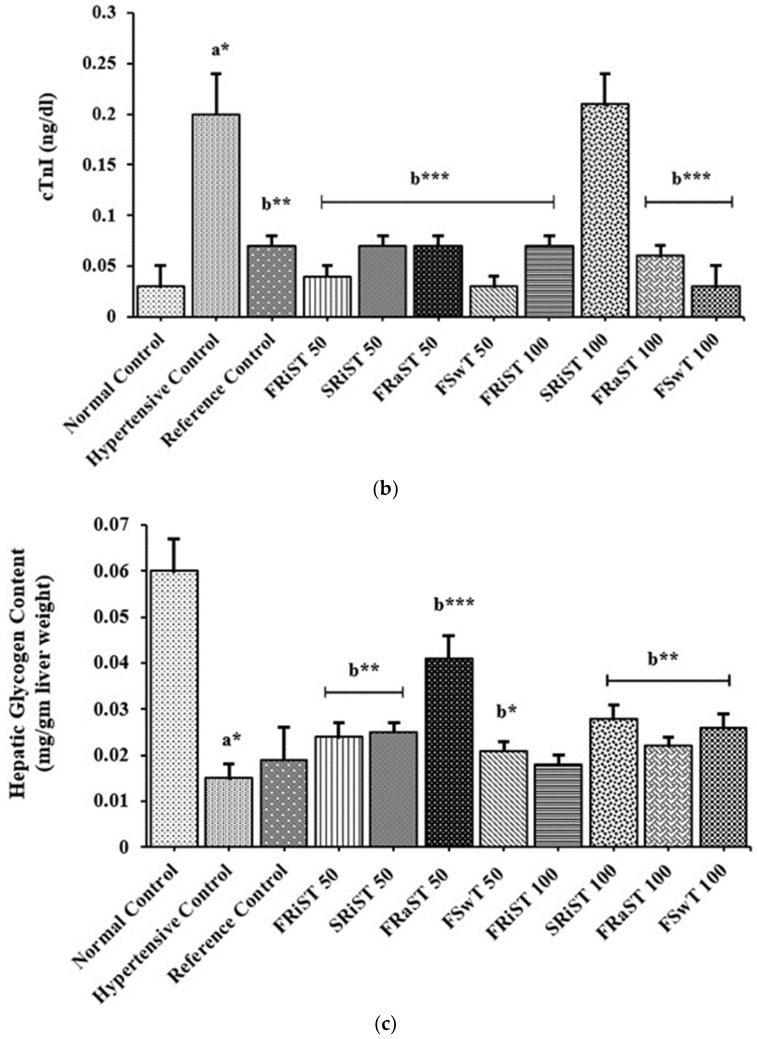
Effects of tamarind products on CRP (**a**), cTnI (**b**), and hepatic glycogen content (**c**). Each bar represents the mean ± SD for *n* = 5 as analyzed by one-way ANOVA followed by *t*-tests. FRiST 50 and FRiST 100 = flesh of ripened sour tamarind aqueous extract at 50 mg/kg BW and 100 mg/kg BW; SRiST 50 and SRiST 100 = seeds of ripened sour tamarind aqueous extract at 50 mg/kg BW and 100 mg/kg BW; FRaST 50 and FRaST 100 = flesh of raw sour tamarind aqueous extract at 50 mg/kg BW and 100 mg/kg BW; FSwT 50 and FSwT 100 = flesh of sweet tamarind aqueous extract at 50 mg/kg BW and 100 mg/kg BW;.CRP = C reactive protein; cTnI = cardiac troponin I. Normal control vs. hypertensive control: *p* < 0.05 = a*; hypertensive control vs. treatment controls: *p* < 0.05 = b*; *p* < 0.01 = b**; *p* < 0.001 = b***. Normal control group = non-hypertensive animals; Hypertensive control group = developed hypertension and left untreated; reference control group = developed hypertension and treated with a reference drug.

The effects of tamarind products on the CRP, troponin I, and liver glycogen levels are summarized in Figure 2. The C-reactive protein of all the groups except the NC group was observed to be increased due to the cholesterol induction. The cholesterol-induced increase in the CRP levels was discovered to be significantly minimized by the treatments of the FRiST50, FRaST50, SRiST50, FSwT100, and SRiST100 groups. Another very crucial biomarker, the serum troponin I level, was observed to be decreased in the treatment groups. The sweet tamarind, at a dose of 50 mg/kg bw (FSwT50), was found to be the most effective at decreasing the troponin I level among the treatments, although all of the treatments significantly reduced the troponin I except SRiST100, which had no effect. The liver glycogen was impacted by almost all of the doses, while the flesh of the raw sour tamarind at a dose of 50 mg/kg/bd (FRaST50) was found to maximally and significantly potentiate the liver glycogen compared to the reference control. At a glance, sweet tamarind, especially SRiST100, was better for increasing the animal liver glycogen than the other forms and doses of tamarind.

### 3.5. Effects of Tamarind Products on Heart Tissue Architecture of Cholesterol-Induced Rat

**Figure 3:**
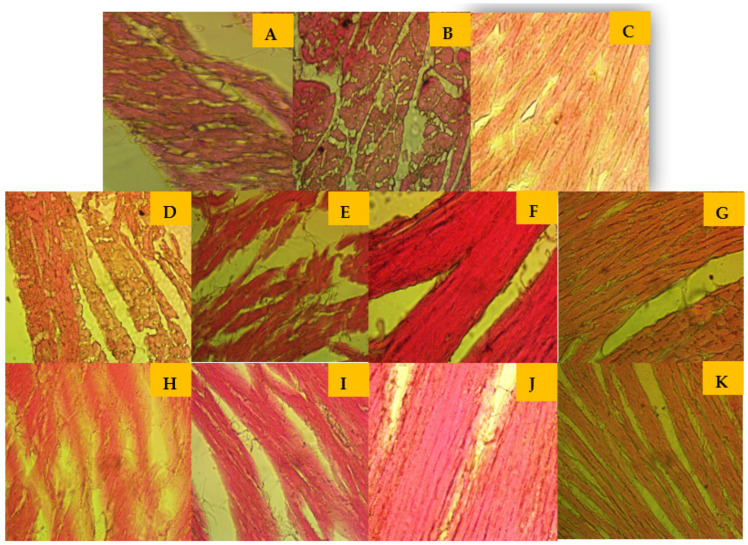
Histopathological images (magnification 10 × 40) of heart tissue of the experimental animals from the groups: (**A**) normal control, (**B**) hypertensive control, (**C**) atenolol control (positive control), (**D**) FRiST50, (**E**) SRiST50, (**F**) FRaST50, (**G**) FSwT50, (**H**) FRiST100, (**I**) SRiST100, (**J**) FRaST100, and (**K**) FSwT100. Normal control group = non-hypertensive animals; hypertensive control group = developed hypertension and left untreated; reference control group = developed hypertension and treated with a reference drug.

A light microscopic examination of the cardiac muscle of the control group showed (Figure 3) a normal myofibrillar structure with striations, a branched appearance, anastomosing cardiac myocytes with central nuclei, acidophilic sarcoplasm, adjacent myofibrils, and a prominent intercalated disc. Photomicrographs of some sections in the cardiac muscle from treated groups, FRiST50, FSwT50, FRaST50, SRiST50, FRaST100, and FSwT100 showed well-maintained separation of the muscle fibers and peripheral nuclei in some fibers’ nuclei. Disarrangement of the cardiac myocyte, cytoplasmic vacuolation, the degeneration of muscle fibers, and vascular infiltration were observed in the slides of groups FRiST100 and SRiST 100.

### 3.6. Phytochemical Contents of the Tamarind Extracts

**Figure 4:**
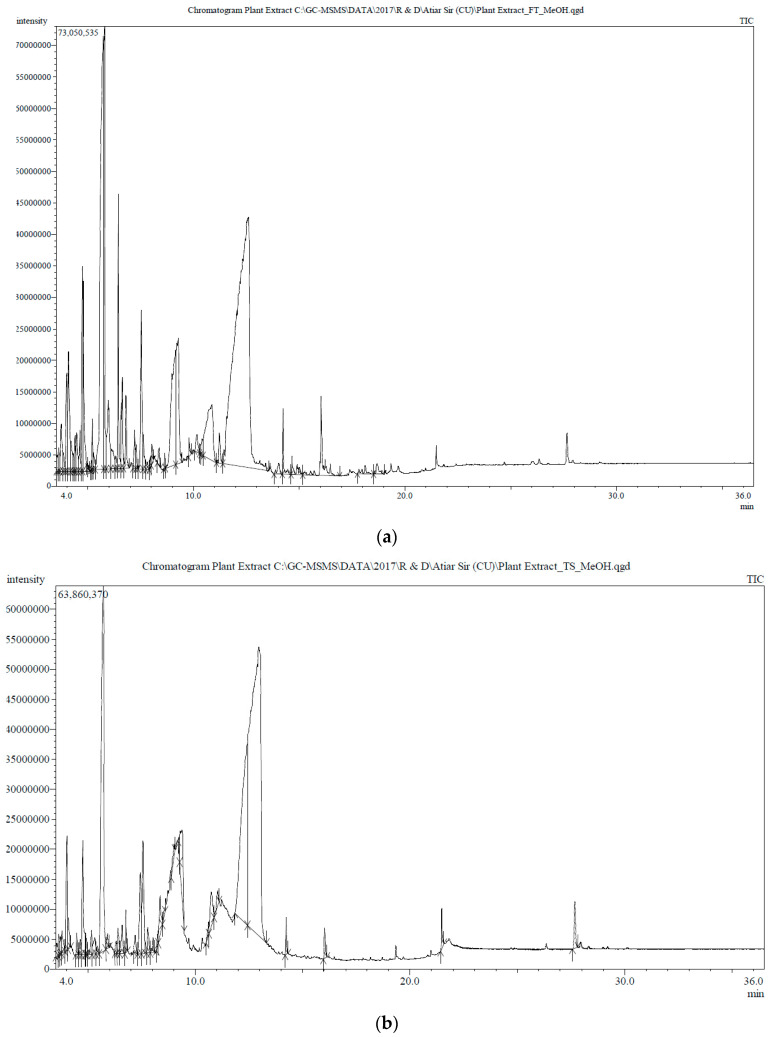
Gas chromatography–mass spectrometry profile of aqueous extract of *Tamarindus indica*; (**a**) flesh, ripened sour, and (**b**) flesh, ripened sweet was obtained by GC-MS with the electron impact ionization (EI) method on a gas chromatograph (GC17A, Shimadzu Corporation, Kyoto, Japan) coupled to a mass spectrometer (GC-MS TQ 8040, Shimadzu Corporation, Kyoto, Japan). The inlet temperature was set at 260 °C, and the oven temperature was programmed as 70 °C (0 min), and then at 10 °C and 150 °C (5 min); 12 °C and 200 °C (15 min); and 12 °C and 220 °C (5 min).

The GC-MS spectra of the sour tamarind and sweet tamarind extracts are shown in Figure 4. The array of compounds is presented in Table 6. Cyclohexanamine, 2-Furanethanol, beta. -methoxy-(S)-, N-3-butenyl-N-methyl, 1-Ethyl-2-hydroxymethylimida Hydrazinecarboxamide, 2-(2-methylcyclohexylidene)- zol, fumaric acid, butyl 3-methylbut-3-enyl ester, 5-(Hydroxymethyl)-2-(dimethoxy methyl) fural, and 3-O-Methyl-d-glucose are noted as the constituents displaying the highest occurrence. Moreover, the data show the presence, in small amounts, of N-glycylglycine, hexadecenoic acid, 1,1-dimethyl ethyl ester, 4H-pyran-4-one, 2,3-dihydro-3,5- dihydroxy-6-methyl-, cis-13-octadecenoic acid, 5-(hydroxymethyl)-2-(dimethoxymethyl)furan.

### 3.7. Impacts of Ligand–Receptor Interactions in In Silico Molecular Docking Analysis

**Figure 5:**
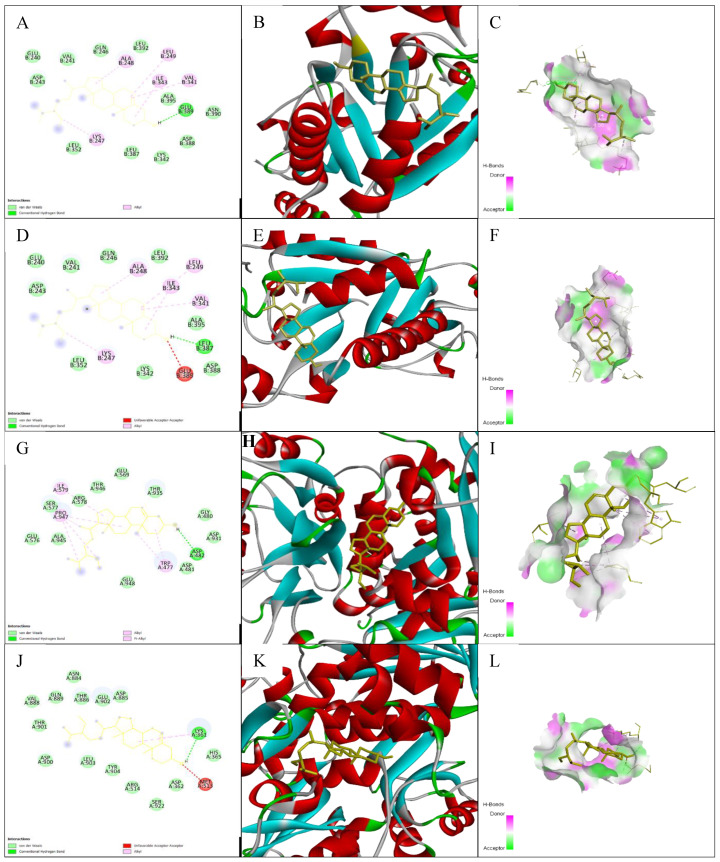

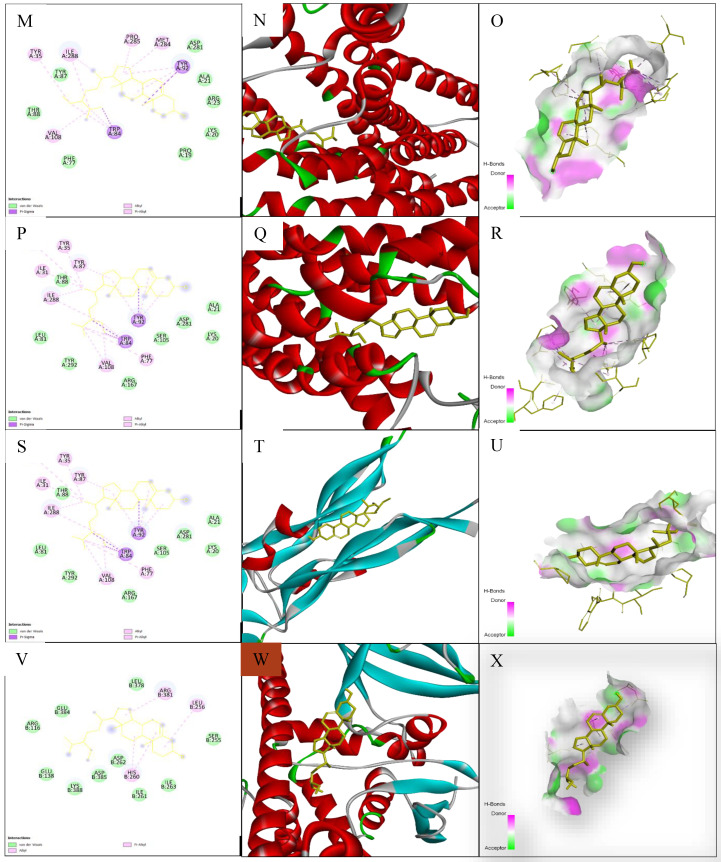
Docking analysis images showing (**A**–**C**) 2D, 3D, and ligand–receptor interaction view of gamma-sitosterol: best binding affinity with tyrosine hydroxylase; (**D**–**F**) 2D, 3D, and ligand–receptor interaction view of gamma-sitosterol: best binding affinity with BETA-1 subunit of the soluble guanylyl cyclase; (**G**–**I**) 2D, 3D, and ligand–receptor interaction view of gamma-sitosterol: best binding affinity with human high-conductance Ca2^+^-gated K^+^ channel (BK Channel); (**J**–**L**) 2D, 3D, and ligand–receptor interaction view of gamma-sitosterol: best binding affinity with nuclear hormone receptor PPAR-gamma receptor; (**M**–**O**) 2D, 3D and ligand–receptor interaction view of gamma-sitosterol: best binding affinity with human angiotensin receptor; (**P**–**R**) 2D, 3D and ligand–receptor interaction view of gamma-sitosterol: best binding affinity with thermolysin; (**S**–**U**) 2D, 3D and ligand–receptor interaction view of gamma-sitosterol: best binding affinity with macrocyclic IL-17A antagonists and (**V**–**X**) 2D, 3D and ligand–receptor interaction view of gamma-sitosterol: best binding affinity with human soluble guanylate cyclase.

The results of the docking analysis of antihypertensive activity through pharmacokinetic properties are shown in Table 7. This study showed that eight major receptors (tyrosine hydroxylase (PDB ID: 1TOH), the BETA-1 subunit of the soluble guanylyl cyclase (PDB ID: 3HLS), human high-conductance Ca^2+^-gated K^+^ channel (BK Channel)(PDB ID: 3NAF), nuclear hormone receptor PPAR-gamma (PDB ID: 3R8A), human angiotensin receptor (PDB ID: 4YAY), macrocyclic IL-17A antagonists (PDB ID: 5HI3), and human soluble guanylate cyclase (PDB ID: 6JT0)) were involved in antihypertensive activity. In the case of the tyrosine hydroxylase (PDB ID: 1TOH), the BETA-1 subunit of the soluble guanylyl cyclase (PDB ID: 3HLS), nuclear hormone receptor PPAR-gamma (PDB ID: 3R8A), and gamma-sitosterol showed a docking score of −7.6 (Figure 5). The docking score was −7.7 for the interaction of gamma-sitosterol with human high-conductance Ca^2+^-gated K^+^ channel (BK Channel) (PDB ID: 3NAF). The interaction of the same compound with human angiotensin receptor (PDB ID: 4YAY), macrocyclic IL-17A antagonists (PDB ID: 5HI3), and human soluble guanylate cyclase (PDB ID: 6JT0) had docking scores of −9.3, −9.1, and −7, respectively (Table 8). The best binding affinity score of gamma-sitosterol was found with guanylate cyclase (PDB ID: 6JT0), which was −9.3.

#### 3.7.1. Impacts on Pharmacokinetic and Toxicological Properties

The pharmacokinetic properties of different compounds were investigated using the QikProp and SwissADME/T (absorption, distribution, metabolism, and excretion/transport) prediction tools. In this study, (1-Ethyl-1H-imidazol-2-yl) methanol, 4H-Pyran-4-one,2-Furanmethanol,5-(dimethoxymethyl)-,7-Tridecanol, glutamine, 1-(2-Thienyl)-propanone, succinic acid, di(but-2-en-1-yl) ester, and 2-(Hydroxymethyl)-2-nitro-1,3-propanediol did not disobey Lipinski’s or Veber’s rules. Hence, these nine compounds exhibited drug-like attributes (Table 9) and were more likely to be orally available as they maximally obeyed Lipinski’s rule and Veber’s rule. On the other hand, D-Mannoheptulose and gamma-sitosterol both had one violation. Furthermore, toxicological properties were also predicted using the admetSAR online server, where the study demonstrated that the compounds are non-carcinogenic except 7-Tridecanol. As a result, ten bioactive ingredients could be investigated as prospective therapeutic candidates with strong oral bioavailability through additional thorough studies, such as an animal model clinical trial.

#### 3.7.2. Impacts on Drug Candidate Filtering

The drug-like characteristics of the observed small molecules were used to screen the main bioactive components in FRiST and FSwT. These requirements included a molecular weight restriction of 500, a minimum of 5 H-bond donors, a maximum of 10 H-bond acceptors, a moriguchi octanol–water partition coefficient value of 5 or less, and an Abbott Bioavailability Score of 0.1 standard values. Surprisingly, 40 detected molecules met the aforementioned requirements and were designated as major bioactive compounds without violating more than one of the previously listed characteristics from which we had selected the top 10 compounds (Table 9).

#### 3.7.3. Common Intersected Targets of Compounds within the GeneCard and SwissTargetPrediction Databases

**Figure 6:**
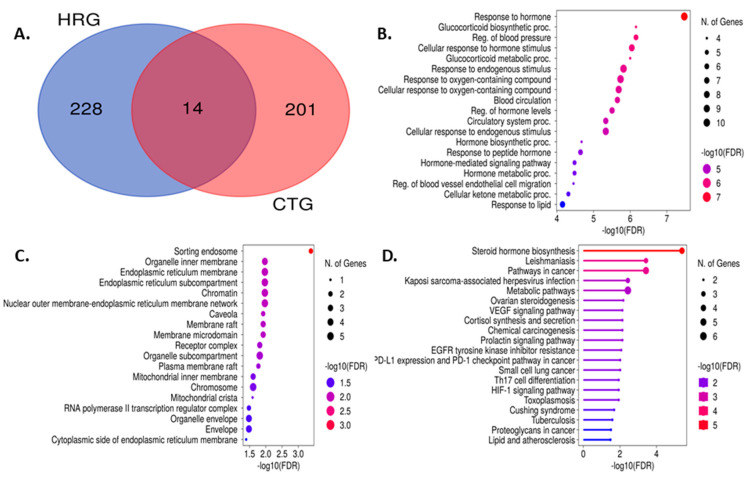
Effects of tamarind on the (**A**) overlapping of genes of hypertension obtained from subtractive analysis of two datasets of 201 and 228 targets; (**B**–**D**) gene ontology (GO) exploration of the compounds in comparison with the targets based on a defined scoring function.

The screened bioactive substances were made to obtain compound-relevant targets from online databases. The PubChem chemical library was used to obtain the SMILES code for each component, which was then inserted into the SwissTargetPrediction database search tool. The elimination of duplicate targets revealed the presence of 228 targets with relevant genes for hypertension from the String database, and 201 targets from the SwissTargetPrediction database, which were connected to 10 compounds. Venn diagram analysis showed that there were 14 common targets between these two datasets (Table S1 and Figure 6A).

DisGeNeT, OMIM, and Malacards were used to access three disease-related public databases, resulting in the acquisition of 3081 disease-related targets. The 238 compound-associated targets were then compared to the culled targets to identify the hypertension targets that were directly related to the different FS and TS compounds according to the confidence level above 10 (Table S2). As a result, 14 shared targets that were directly related to the hypertension and FRiST and FSwT compounds were identified, of which 10 molecules were closely related to typical hypertension targets. The 10 compounds included (1-Ethyl-1H-imidazol-2-yl)methanol, 4H-Pyran-4-one, 7-Tridecanol, D-Mannoheptulose, gamma-sitosterol, glutamine, 1-(2-Thienyl)-1-propanone, succinic acid, di(but-2-en-1-yl) ester, and 2-(Hydroxymethyl)-2-nitro-1,3-propanediol. These substances were produced by the interaction of genes associated with hypertension and the target genes in the FRiST and FSwT substances.

### 3.8. Impact on PPI Network Analysis of 14 Common Targets

**Figure 7:**
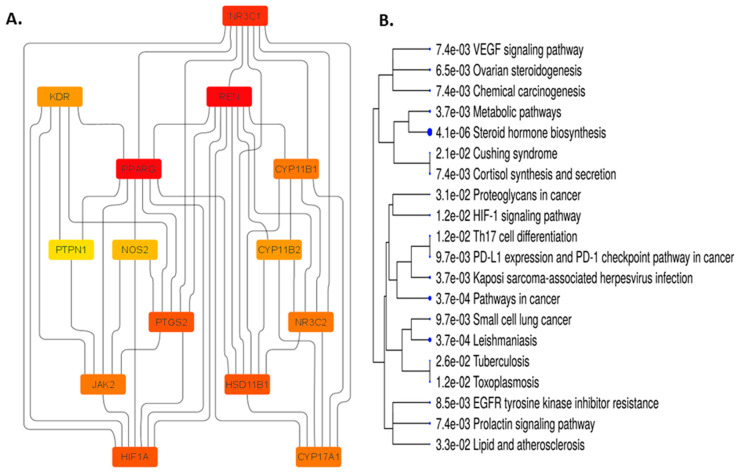
Interaction plot of proteins (PPI) of common interacting genes utilizing the (**A**) degree algorithm: 14 nodes representing 14 potent proteins with interconnections (edges) between them as a narration of their multi-pathway involvement, and (**B**) hierarchy tree of pathways: different pathways involved in hypertension where steroid hormone biosynthesis showed the highest degree value.

We added the 14 common targets to the STRING database to create a network among them to discover the possible mechanistic roles of FRiST and FSwT in managing hypertension. The STRING method expressed the 14 joined nodes by creating 44 edges at the same time (Figure 7A). We then analyzed the network in Cytoscape using a degree value method and cytoHubba applications to examine the crucial main target in the network. The number of degrees for each target was specified as the number of edges connecting to the relevant target nodes. Notably, the best target in the network was identified by a higher degree value. *NR3C1*, *REN*, *PPARG*, and *CYP11B1* were identified as critical targets (36-degree value) in the network for hypertension progression because of this conformity (Figure 7B). 

#### 3.8.1. Impact on the Enrichment Analysis of 14 Common Targets

Through pertinent targets’ molecular function (MF), the biological process (BP) in which it participates, and its cellular localization, the GO and KEGG pathway assessment accurately depicts the intersected 14 common targets involved in the hypertension functional process (Tables S3–S5). We examined the GO and KEGG pathways using the online application “ShyniGO 0.77”. According to the percentage of targets that were enriched in the top 10 MF, BP, and chemical categories, we were able to determine the top 10 MF, BP, and chemical contents for GO (Figure 6B–D). MF is primarily involved in oxidoreductase activity, acting on paired donors with incorporation or reduction, steroid hydroxylase activity, Hsp90 protein binding, steroid hormone receptor activity, nuclear receptor activity, ligand-activated transcription factor activity, E-box binding, and NADP binding. 

#### 3.8.2. Glucocorticoid Biosynthetic Process

**Figure 8:**
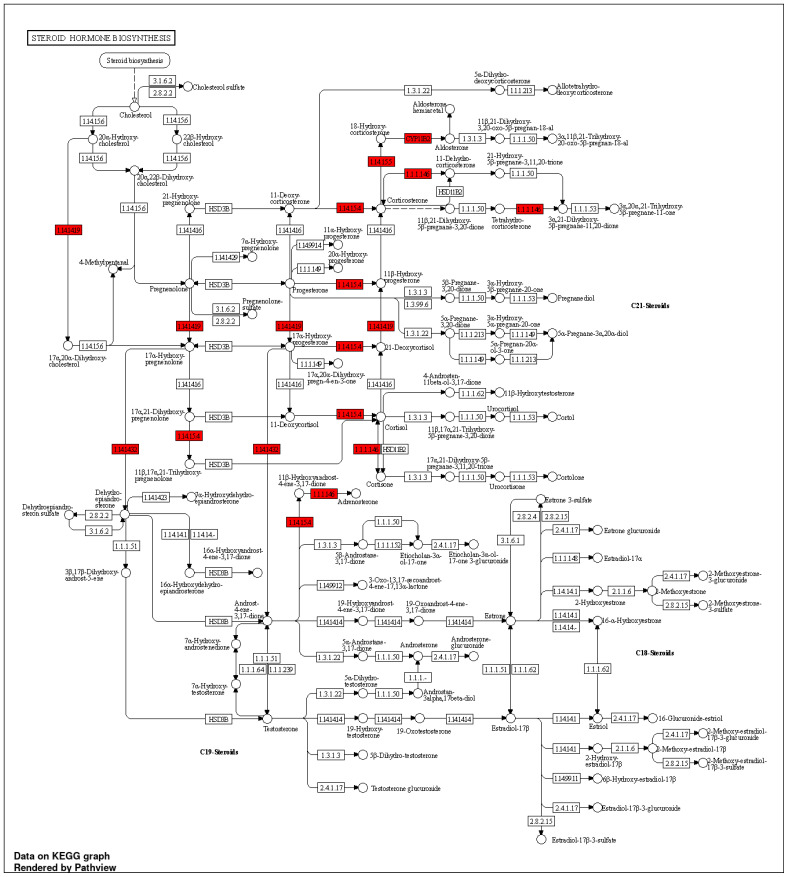
Steroid hormone biosynthesis pathway. The genes involved in the process may have significant potential to be involved in the hypertension mechanism. The 14 targeted genes are highlighted in red color and the subducing pathways are the most significant pathways related to hypertension.

The glucocorticoid metabolic process, hormone biosynthetic process, regulation of blood vessels, endothelial cell migration, regulation of blood pressure, hormone-mediated signaling pathway, hormone metabolic process, cellular ketone metabolic process, and blood circulation process were the BPs in which the targets notably participated. However, the aforementioned processes happened in the top 10 CLs, which were sorted into the endosome, mitochondrial crista, cytoplasmic side of the endoplasmic reticulum membrane, caveola, plasma membrane raft, RNA polymerase II transcription regulator complex, membrane raft, membrane microdomain, and receptor complex. Similar to this, 21 KEGG pathways (Table S6) were found at a threshold level of *p*-value 0.05 in the KEGG pathway enrichment analysis performed using the ShyniGO 0.77 online tool on the 14 possible treatment targets for hypertension in steroid hormone biosynthesis (Figure 8). 

### 3.9. Molecular Dynamics Simulations

**Figure 9:**
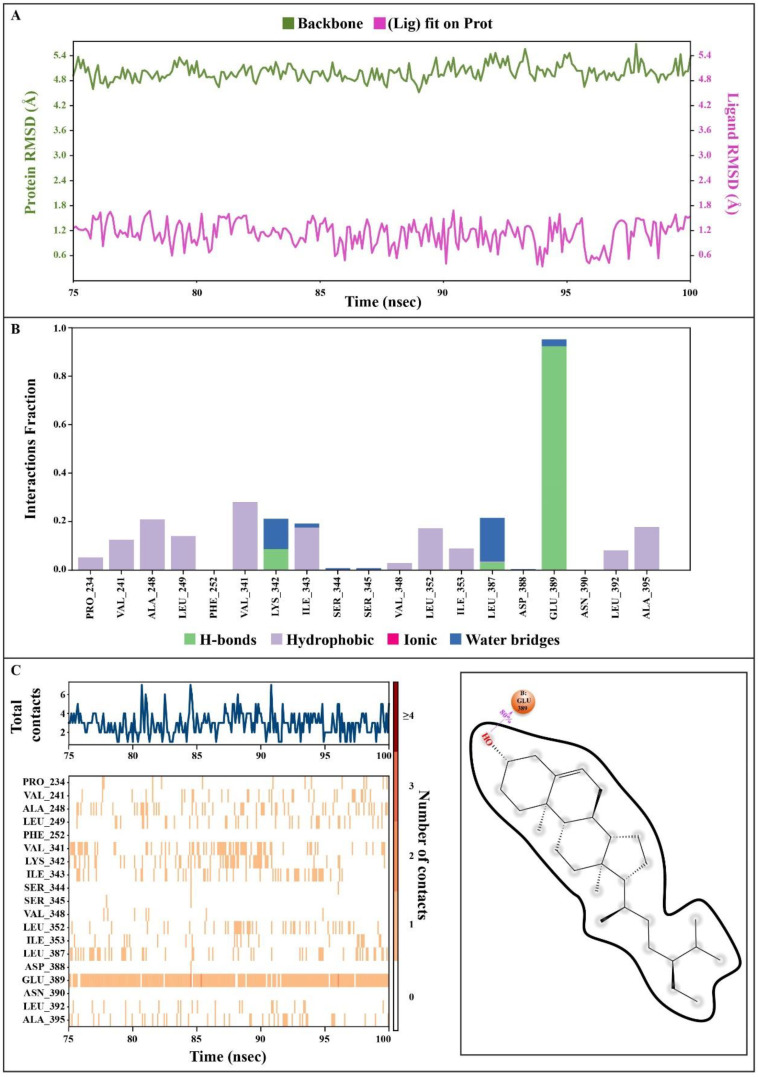
Diagram depicting simulation interactions of a protein–ligand complex under in vivo mimic circumstances. (**A**) Protein–ligand RMSD plot: the left Y-axis represents the protein RMSD, and the right Y-axis represents the ligand RMSD. (**B**) Histogram of protein–ligand interactions classified as hydrogen bonds (green), hydrophobic (purple), ionic (magenta), and water bridges (blue). (**C**) A depiction of the interactions and contacts on a timeline: the left top panel depicts the total number of specific contacts made by the protein with the ligand during the equilibrated trajectory (from 75.00 to 100.00 ns), whereas the left bottom panel depicts which protein residues interacted with the ligand in each trajectory frame. According to the scale to the right of the plot, certain residues made more than one particular contact with the ligand, which is indicated by a deeper shade of orange. The right panel depicts a schematic of precise ligand atom interactions with the protein residues. Interactions that occurred more than 30.0% of the time in the chosen trajectory (75.00 to 100.00 ns) are displayed.

**Figure 10:**
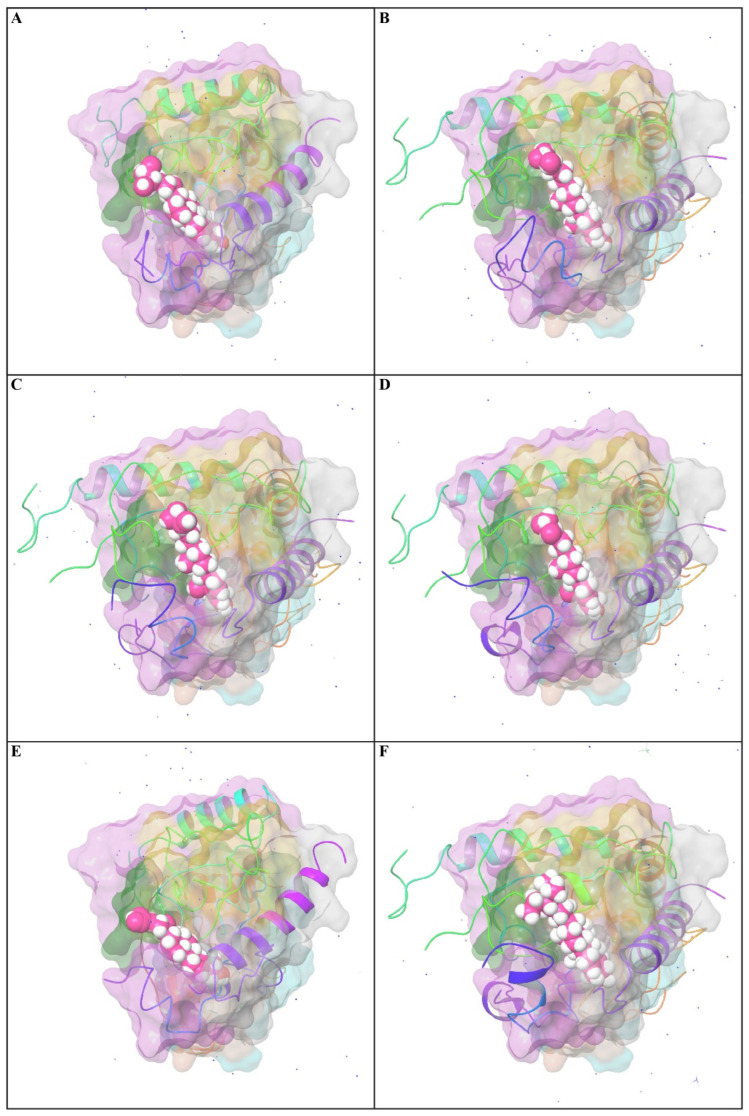
The position of a ligand inside the pocket side during the selected trajectory (from 75.00 to 100.00 ns). (**A**) at 75 ns, (**B**) at 80 ns, (**C**) at 85 ns, (**D**) at 90 ns, (**E**) at 95 ns, (**F**) at 100 ns.

The dynamic kinematics of the docked complexes were subsequently examined by molecular dynamic simulations at 100 ns using the Desmond module of Schrödinger’s suite to study the stability of the protein–ligand combinations under in vivo mimic conditions. The results are shown in Figure 9 and Figure 10 and Supplementary Video S1 (The video shows molecular dynamics simulations of the whole protein–ligand complex under in vivo mimic conditions. https://drive.google.com/file/d/1UefhKZrJk1APbYLW8cPWazeCONILLsle/view?usp=share_link, accessed on 11 June 2023).

#### 3.9.1. Protein Root Mean Square Deviation (P-RMSD)

The P-RMSD was used to calculate the difference in a protein’s backbones from its initial structural conformation to its final position. The deviations induced during the simulation of the protein can be used to estimate its stability [27]. It was computed for each frame of the trajectory. In Figure 9A, the left Y-axis demonstrates a protein’s RMSD evolution. All protein frames were first aligned on the reference frame backbone before calculating the RMSD based on the atom selection. The results show that the P-RMSD values of the protein–ligand complex were stable between 75.00 and 100.00 ns, with the highest value being 5.68 and the lowest value being around 4.52, showing that the system equilibrated during this period.

#### 3.9.2. Ligand Root Mean Square Deviation (L-RMSD)

The L-RMSD (aligned on the ligand) demonstrates the ligand’s stability (Figure 9A, right Y-axis). This RMSD value measures the internal fluctuations of the ligand atoms. ‘Lig fit Lig’ illustrates the RMSD of a ligand that was aligned and assessed on only its reference conformation in this plot. During the MD simulation, the highest value of the L-RMSD was 1.69, while the lowest value was around 0.34.

#### 3.9.3. Protein–Ligand Contacts

Throughout the simulation, protein interactions with the ligand could be well observed. During the simulation, the stacked bar charts showed that the complex had H-bonds, hydrophobic interactions, and water bridges (Figure 9B). The number of individual connections produced by proteins with the ligands throughout the selected trajectory (75.00 to 100.00 ns) was in the range of 0 to 7 contacts, according to a timeline depiction of the interactions and contacts (Figure 9C). Furthermore, the findings revealed that glutamic acid at protein residue position 389 (Glu389) was the protein residue that interacted with the ligands most frequently (Figure 9C). A schematic of the precise ligand atom interactions with these protein residues is also shown in Figure 9C, at the right.

#### 3.9.4. Position of a Ligand Inside the Pocket Side

The position of a ligand inside the pocket side during the MD simulation’s equilibrated system (75.00 to 100.00 ns) is shown in Figure 10 and Video S1. The snapshot of the protein–ligand complexes every five ns illustrate that the ligand was located at the same pocket site (Figure 2). The interactions and behavior of the ligand inside the pocket side during this equilibrated system are displayed in Video S1.

## 4. Discussion

The results of the present study reveal the antihypertensive effect of the extracts of ripened sour *T. indica* (FRiST), the flesh of raw sour *T. indica* (FRaST), the flesh of ripened sweet *T. indica* (FSwT), and the seeds of sour *T. indica* (SRiST), and their cooperation in normal and cholesterol-induced hypertensive rats in a dose-dependent fashion. A cholesterol-rich diet and high-fat diets are linked to dyslipidemia, which is considered a major risk factor for hypertension.

Polyphenols are a group of bioactive compounds, with more than 7000 chemical entities present in different cereals, fruits, and vegetables. These natural compounds, which impact the total phenolic and flavonoid contents as well as the total antioxidant capacity, possess many OH groups, which are largely responsible for their strong antioxidative and anti-hypersensitive properties [28]. Polyphenols have attracted scientific interest for their beneficial effects in preventing oxidative stress-induced endothelial dysfunction by increasing eNOS activity, which has been shown to scavenge ROS, inhibit NADPH and xanthine oxidases, and chelate metals, which altogether increase the NO bioavailability, with an antihypertensive effect [29]. Proanthocyanidins, the largest and most ubiquitous plant polyphenolics, are reported to serve as a novel antihypertensive therapy by modulating the cardiovascular disease risk markers, such as blood pressure and blood lipids [30,31,32]. Based on the existing research, the antihypertensive effect of the polyphenolic content is related to the activation of the nitric oxide system [33], the regulation of endothelial function, and the inhibition of angiotensin I-converting enzyme (ACE) activity [34], which is required for the therapeutic intervention to control CVD-associated hypertension. Fernandez et al. evaluated the effects of the polyphenolic contents on ACE and showed that they could significantly inhibit the activity of ACE and NADPH oxidase, which might be one of the potential cardioprotective mechanisms [35,36]. Our study showed the highest presence of polyphenolic contents in the FRaST aqueous extracts, while other prior studies have revealed higher polyphenolic contents in FRiST. However, further investigation might be needed for the quantitative polyphenolic estimation in the methanolic extracts of FRiST, FRaST, FSwT, and SRiST to evaluate their ACE inhibition capacity. Polyphenolics may also indirectly influence blood pressure by modulating inflammation and oxidative stress to reduce more than one CVD risk factor evident in animal studies [37,38]. Previous studies have confirmed that polyphenolics can display anti-inflammatory effects by significantly downregulating the expressions of TNF-α, MCP-1 and IL-6 in high-fat-diet-fed mice. Kanamoto et al. found that PCs significantly inhibited the expression levels of TNF-α, MCP-1 and IL-6 in high-fat-diet-fed mice [39,40]. Therefore, polyphenolic supplementation may be a useful treatment for hypertensive patients and a preventive measure for prehypertensive and healthy subjects [41]. Furthermore, bioflavonoids show vasodilator effects in isolated aortae stimulated with noradrenaline, KCl, or phorbol esters, and these effects are independent of the presence of endothelium [42]. Thus, this direct vasodilator effect and antioxidant property might contribute to its antihypertensive effects observed in the present study. Further investigation is needed to evaluate the antioxidant activity of the four sample water extracts [43].

High dietary cholesterol has been shown to increase plasma cholesterol and may speed up the development of aortic atherosclerosis [44]. Numerous studies have shown that lowering cholesterol with diet or medication can reduce morbidity and mortality from CVD in the future. Based on this, significant efforts have been undertaken to lower the risk of CVD through the control of cholesterol, and therefore, the therapeutic advantages of plant foods have been the subject of several in-depth dietary studies (Yokozawa T) (Yokozawa T) [45]. A study by Shivshankar and Shyamala Devi reported that rats supplemented with 10% *T. indica* pulp aqueous extract demonstrated significantly reduced body weight after 2 weeks of treatment. The hypocholesterolemic effects of *T. indica* pulp fruit extracts were also reported in 2006 by Martinello et al. [46]. Tamarind extract administration with cholesterol was found to inhibit the gaining of body weight in all extract groups except SRiST100. When compared to NC and PC, FRiST50 and FRaST50 significantly reduced body weight, confirming prior findings. The use of male rats in our experiment is significant because hypertension is linked with the renin–angiotensin–aldosterone system (RAAS), and it is evident that renal sympathetic nerve activity is less excitable and more easily repressed in females than males [47,48].

The crucial risk factor for CVD includes a low level of HDL cholesterol, which plays a direct role in the atherogenic process, and a low level of HDL cholesterol and an increased risk of CVD is well established [49]. In the present study, apart from its weight-reducing ability, aqueous extract supplementation was observed to significantly decrease the levels of total cholesterol, total TG, and LDL, and increase the HDL level by more than 50% in the plasma of the treatment group, which reversed the effects of high-fat diet consumption alone. The elevation of the HDL concentration was found to be dose-dependent, where the group treated with FRaST 100 showed the highest increment, followed by FRiST extract at 100 mg/kg and FSwT extract at 100 mg/kg. Similar results were obtained by Martinello et al. [50]. The increase in HDL may be explained by the counteracting LDL oxidation, which promotes the reverse cholesterol transport pathway by inducing an efflux of excess accumulated cellular cholesterol or by the transition metal ion-based inhibition of LDL oxidation [51].

A dose-dependent administration of aqueous tamarind extracts also provided a beneficial effect to the reduction of the total cholesterol, triglycerides, LDL cholesterol, and VLDL cholesterol. The lipid-lowering potential of the extract may be attributed to the presence of phytochemical constituents, such as flavonoids, saponins, and tannins [52,53]. Flavonoids are reported to decrease LDL cholesterol and increase HDL cholesterol concentrations in hypercholesteraemic animals [54]. Saponins are reported to inhibit pancreatic lipase activity in high-fat-diet-fed mice, leading to greater fat excretion due to reduced intestinal absorption of dietary fats [52]. In this experiment, FRiST50 in the experimental group was found to be better for minimizing TC, TG, and LDL, and the enriched content of flavonoids had a positive impact on this action.

An interrelationship between the functional integrity of the liver and the development and maintenance of hypertension is being increasingly recognized [52]. An absence of experimental and clinical hypertension with liver disease has been noted [55]. Alanine aminotransferase and aspartate aminotransferase, respectively localized in the hepatocellular cytosol and mitochondria, are the most specific markers of hepatic injury [56]. In the present study, the hepatic enzymes (ALT and AST) were significantly lower in the antihypertensive treatment groups, except in the FRaST50, FSwT50, FRiST100, and SRiST100 groups, which reflected a dose-dependent regulation of hypertension in the rat model. The serum level of ALP was higher in the antihypertensive subjects than in the normotensive animal in a dose-dependent fashion. When different varieties and parts of Tamarindus were considered, elevated ALP levels were more likely to be found in the cholesterol-induced hypertensive rate of the FRiST50, FRaST50, FSwT50, FRaST100, FSwT100 groups, with low risk of hypertension. A high prevalence of elevated levels of ALT and GGT demonstrated a higher risk for hypertensive females and males than their normotensive counterparts. A similar result was found in a previous study, which reported a high prevalence of elevated ALT in the hypertensive group compared to the normotensive group [57]. There is no simple explanation for why a serum ALT showed an independent association with hypertension in the Bangladeshi population. One possibility may be that hypertensive individuals develop non-alcoholic fatty liver disease (NAFLD) after a long period of elevated blood pressure [58]. The postulated mechanism is that increased blood pressure activates pro-inflammatory responses, such as TNF-α and interleukin adiponectin and leptin, which contribute to hepatotoxicity [59]. In parallel, oxidative stress is documented to be associated with hypertension [60] and antioxidant enzyme gene polymorphisms, including a few of the glutathione-S-transferase genes, have been reported to be correlated with the risk of hypertension in general adults [61,62].

The activity of the gluconeogenic enzyme, glucose-6- phosphatase, is usually enhanced during diabetes. After extract administration, the blood glucose levels dropped while the amount of liver glycogen increased. This may have been due to the mobilization of blood glucose into the liver glycogen reserve [63,64]. Ramsay, in 1977, discovered that 15% of experimental male hypertensive patients had abnormal liver function tests, suggesting a link between abnormal liver function tests and hypertension [65]. Animal models have also suggested a potential role for angiotensin II in the progression of NAFLD to hepatic fibrosis [66], and the use of angiotensin II type 1 receptor antagonists has been shown to reduce this progression [67]. The high liver glycogen level in cholesterol-induced hypertensive rats may be due to either an increase in gluconeogenesis or hyperglycemia due to 18 h of fasting before testing. In this study, in all treated groups except FRaST50, the reversion of the liver glycogen towards normal levels may have been due to its activating effect on glucokinase and glycogen synthetase.

The C-reactive protein (CRP), a prototypical acute-phase reactant, is one of the most widely known biomarkers of cardiovascular disease. The circulating levels of CRP are clinically used to predict the occurrence of cardiovascular events and to aid in the selection of therapies based on more accurate risk assessment in individuals who are at intermediate risk. Hypertension has a positive correlation with the CRP level. Cholesterol administration elevated the CRP level. Antihypertensive drug and extract administration along with cholesterol lowered the CRP level. The extract groups of sweet *T. indica* (FSwT100), ripened sour *T. indica* (FRiST50), and raw *T. indica* (FRaST100) significantly decreased the CRP levels compared to NC and PC [68].

Previous studies have suggested that chronic subclinical myocardial damage, detected by elevated the troponin I level, may precede the development of hypertension in the general population and that this novel biomarker of cardiac damage may have utility in identifying people at future risk for hypertension and hypertensive end-organ damage [69]. In addition, the histopathology of cardiomyocytes was also performed to corroborate the findings of the biochemical investigation [70]. Our study also ameliorates the previous finding that myocardial damage along with troponin I level elevation is carried out synergistically. The tissue architecture of the histopathological analysis reflected the partial amelioration of different cardiac sections that changed through the hypertension-producing treatment. Histopathological observations showed less damage in the tamarind extract-treated group than in the cholesterol-induced group, and sometimes, it was better than in the positive control [71]. As a result, it can be stated that the *Tamarindus indica* extract was highly effective in preventing cholesterol-induced hypertension in rats. Histopathological analysis of the heart myocyte and troponin I levels revealed that myocyte disruption and myofibril infiltration appeared in the SRiST100 group, meanwhile, troponin I was significantly elevated compared to other extracts and control groups. Further study is needed to reveal the composition difference of SRiST extract compared to the other extract groups. In the present study, the findings in the cardiac tissues were found to be less in the FRiST group compared to the PC group. *T. indica* is enriched with antioxidant compounds such as flavonoids and vitamins C and E [72]. The antioxidant activity of the extract, which occurs through its free-radical scavenging activity, may have prevented oxidative damage to the myocardium in the cholesterol-induced hypertensive rats through acetylcholine esterase inhibitory (ACE inhibition) actions [73]. Furthermore, the antioxidant activity of quercetin contributed to *T. indica* extract would be helpful to manage glucose uptake and the glucose-induced increased levels of mitochondrial reactive oxygen species (ROS) linked to hyperglycemia [67,74]. The results of the present study show that treatment with *T. indica* extract, except for SRiST100, decreased cardiac damage in cholesterol-induced hypertensive rats.

Oxidative reactions play an important role in plaque progression and instability, and the oxidation of LDL is the main event in the pathogenesis of atherosclerosis. Iron-dependent LDL may become critical when the progression of atheroma towards end-stage plaques leads to the liberation of iron ions, which mediate LDL oxidation by GSH hydrolysis production. In our study, we found a few compounds that were directly related to the antihypertensive activity. Among the compounds, a potentially useful zwitterionic buffer in the physiological pH range (6.0–8.5) is a novel class of glycylglycine amides that was discovered to have value as antiarrhythmic agents [73]. Additionally, glycylclycine was reported to work as an acceptor in the catabolism of GGT, which is raised in serum in cardiovascular (CV) mortality [75]. Among others, decanoic acid, 3-methyl- is reported to display antihypertensive activity through its antioxidant and anti-inflammatory action [76].

Molecular docking analyses are widely used to explore ligand–target interactions to identify the appropriate drug target for therapeutic innovation. They shed more light on the likely methods of action and binding manner of various proteins’ binding pockets [77]. Molecular docking in our study was used to further verify the antihypertensive action of T. indica through its ten lead compounds. The compounds were docked against eight targeted receptors described in the methodology section. Among the compounds, gamma-sitosterol had the strongest binding interactions. The title compounds’ anti-heart failure action could be mediated via binding or inhibiting tyrosine hydroxylase, BETA-1 subunit of guanylyl cyclase, BK channel, AT1 receptor antagonist, AT1 receptor antagonist combination with PPAR agonist, thermolysin, macrocyclic IL-17A antagonists, and human soluble guanylate cyclase. However, gamma-sitosterol has shown the best binding efficacy with the soluble guanylyl cyclase (GC-1) receptor, which supports vascular function by catalyzing the conversion of GTP to cGMP via the NO/GC-1/cGMP pathway [78]. The cGMP pathway stimulates protein kinase G, which phosphorylates a variety of substrates to cause vasodilation and the prevention of platelet aggregation and adherence to the artery wall, among other actions. Dysfunction in the NO/GC-1/cGMP pathway has been linked to several vascular disorders via reactive oxygen species (ROS) [79].

The process of drug development requires the toxicity assessment of novel compounds [80]. The pharmacokinetic properties of *T. indica* phytocompounds were tested by Lipinski’s rule of five, which states that orally administered drugs should have a molecular weight ≤ 500 amu, hydrogen bond acceptor sites ≤ 10, hydrogen bond donor sites ≤ 5, and a Log P lipophilicity value of ≤ 4.15, and Veber’s rule of two (number of rotatable bonds ≤ 10, topological polar surface area ≤ 140). The violation of these rules by any drug or phytoconstituent will lead to disqualifying its oral bioavailability as a good drug. The gamma-sitosterol of *T. indica* demonstrated the worthiest toxicokinetics, ensuring its good oral bioavailability. Preclinical toxicity testing of GCMS compounds was studied in this work, utilizing the admetSAR online server, and the results show that all compounds are nontoxic and noncarcinogenic.

Network pharmacology (NP), which uses computational power to systematically catalogue the molecular interactions of a drug molecule in a living cell, is an emerging attempt to understand drug actions and interactions with multiple targets [81]. Protein–protein interaction (PPI) revealed the possible targets of identified compounds from the extract, which are correlated with hypertension-related pathways. CYP17A1 is an enzyme that contributes to the synthesis of cortisol and aldosterone, which are important for controlling blood pressure. Studies have revealed that *CYP17A1* gene variants may be linked to elevated blood pressure and a higher risk of hypertension [82]. The aldosterone-mediated regulation of blood pressure is mediated by the mineralocorticoid receptor, which is encoded by the NR3C2 gene. According to studies, specific *NR3C2* gene variants may have a role in the emergence of hypertension [83]. The *VEGF* receptor 2, which is involved in controlling blood vessels, is encoded by the *KDR* gene. Blood pressure and the likelihood of developing hypertension have both been linked to higher *KDR* expression [84]. The *NOS2* gene is relevant for the nitric oxide-producing inducible nitric oxide synthase (iNOS) enzyme. Nitric oxide aids in blood pressure control and blood vessel relaxation. Blood pressure and hypertension have been linked to NOS2 deficiency [85]. The protein tyrosine phosphatase gene *PTPN1* modulates blood vessel tone, which in turn regulates blood pressure. According to studies, PTPN1 gene variants may have a role in the emergence of hypertension [86]. The JAK2 gene encodes a protein that participates in signaling pathways that control blood pressure. According to studies, there may be a link between JAK2 gene variants and an increased risk of hypertension [68]. The COX-2 enzyme, which is essential for the generation of prostaglandins, is encoded by the PTGS2 gene. Changes in prostaglandin levels can contribute to the development of hypertension, as prostaglandins regulate blood pressure [69]. Renin, an enzyme involved in the control of blood pressure and fluid balance, is encoded by the gene REN. Renin expression has been associated with elevated blood pressure and a higher risk of hypertension [70]. HSD11B1 is an enzyme that participates in the metabolism of the hormone cortisol, which can have an impact on blood pressure. According to studies, hypertension risk may be increased by polymorphisms in the HSD11B1 gene [87]. Aldosterone, a hormone that controls blood pressure, is produced by the enzyme CYP11B2 in the body. According to studies, people who have CYP11B2 gene variants may be at a higher risk of developing hypertension [88]. A gene called PPARG encodes a protein that controls insulin sensitivity and blood pressure. According to studies, hypertension risk may be increased by polymorphisms in the PPARG gene [89]. 

The emergence and progression of hypertension have been linked to the genes HIF1A, CYP11B1, and NR3C1. The hypoxia-inducible factor 1 (HIF1A) gene controls how much oxygen is present in the body. This gene’s impact on blood pressure control and angiogenesis has been found to contribute to the onset of hypertension and cardiovascular disease [90]. The gene CYP11B1 encodes a cytochrome P450 enzyme, which is necessary for the manufacture of hormones, notably, the stress hormone cortisol. Through its impact on cortisol levels, which can affect blood pressure regulation, this gene has been associated with hypertension [91]. The glucocorticoid receptor, a crucial regulator of the stress response and cortisol levels, is encoded by the nuclear receptor subfamily 3 group C member 1 (NR3C1) gene. Studies have revealed that NR3C1 polymorphisms can affect blood pressure regulation and cardiovascular function, and variations in this gene have been linked to an increased risk of hypertension [92].

It is crucial to keep in mind that although these genes have been linked to the control of blood pressure and the onset of hypertension, the relationship is complicated and may be altered by several variables, including a person’s lifestyle and environment. To fully comprehend how these genes contribute to hypertension, more investigation is required.

Several genes, including *CYP17A1*, *CYP11B2*, *HSD11B1*, *CYP11B1*, and *NR3C2*, which are implicated in the control of hypertension, regulate the synthesis of steroid hormones, such as glucocorticoids, mineralocorticoids, androgens, and estrogens. Blood pressure can be influenced by the number of steroid hormones generated, and differences in the expression of these genes have been linked to a higher risk of hypertension [93]. Leishmaniasis has been connected to the emergence of hypertension and cardiac disease. Leishmaniasis is a parasite disease. The parasite’s impact on the immune system, which can cause inflammation and alterations in blood pressure, is probably to blame for this. Numerous genes, including *PTPN1* and *NOS2*, have been linked to the emergence of hypertension in leishmaniasis patients [94,95]. The ovaries play a role in the synthesis of estrogen and other hormones, and differences in the expression of ovarian steroidogenesis genes, such as *CYP17A1* and *CYP11B2*, have been linked to a higher risk of hypertension [96]. Additionally, the *HSD11B1* gene has been linked to the control of blood pressure and estrogen levels. The VEGF signaling pathway is connected to hypertension and is involved in the control of angiogenesis, and the formation of new blood vessels. Variations in how this pathway’s genes, including KDR, are expressed have been linked to a higher risk of hypertension [97]. Cortisol, a glucocorticoid hormone, controls blood pressure and the body’s reaction to stress. Several genes, including *CYP11B2*, *NR3C2*, and *HSD11B1*, control the synthesis and secretion of cortisol. Variations in the expression of genes implicated in this system, such as PRL, have been linked to an increased risk of hypertension. This pathway is important in the control of breastfeeding and hormone synthesis [98]. Resistance mechanisms to EGFR tyrosine kinase inhibitors are a class of cancer therapies that focus on the epidermal growth factor receptor. Changes in several genes, including *JAK2*, *PTGS2*, and *HIF1A*, which have also been connected to the emergence of hypertension, have been linked to resistance to these inhibitors. Gamma-sitosterol, a phytosterol, resembles cholesterol. Phytosterols may lower cholesterol, improve cardiovascular health, and reduce the incidence of some cancers. Angiotensin regulates blood pressure and fluid balance. It affects angiotensin receptors. Both AT1 and AT2 receptors regulate blood pressure and fluid balance [99]. Gamma-sitosterol and angiotensin receptors are poorly explored. Gamma-sitosterol may alter angiotensin receptor activation, which may improve blood pressure and cardiovascular health. Research on the relationship between gamma-sitosterol and thermolysin is scarce. Thermolysin is one of the proteases that may be able to be inhibited by phytosterols, particularly gamma-sitosterol, according to certain studies [100]. Potential therapeutic advantages of thermolysin and other protease inhibitions include the treatment of cardiovascular disease, cancer, and inflammation.

Studies using MD simulations are thought to be useful for determining the relative stability and dynamic properties of ligand–target complexes. Additionally, MD simulations are more effective than static images produced by molecular docking and the mechanical energy minimization technique for studying the complicated conformation space [101,102]. In the present study, we explored the binding affinity-based biological stability of the drug-likely antihypertensive molecules from *T. indica* to macromolecular receptors, such as tyrosine hydroxylase (PDB ID: 1TOH), BETA-1 subunit of the soluble guanylyl cyclase (PDB ID: 3HLS), human high-conductance Ca^2+^-gated K^+^ channel (BK channel) (PDB ID: 3NAF), nuclear hormone receptor PPAR-gamma (PDB ID: 3R8A), human angiotensin receptor (PDB ID: 4YAY), macrocyclic IL-17A antagonists (PDB ID: 5HI3), and human soluble guanylate cyclase (PDB ID: 6JT0). In our molecular simulation study, gamma-sitosterol was found to be a common bioactive molecule that interacted with the majority of the selected target proteins, especially guanylate cyclase, which showed the lowest ligand RMSDs values compared to those of their respective proteins. The dynamic behaviors of the ligand–receptor complexes confirm significant ligand/pocket accommodation, successful complex stability, and MD simulation convergence [103]. The protein’s collective dynamic motion/behavior was examined from MD simulation trajectories as part of further validation and monitoring of MD simulation convergence. Gamma-sitosterol’s conformational stability at the guanylate cyclase, as determined by an active site and MD modeling, supports the substance’s potential for use as a medication.

## 5. Conclusions

This research was focused on the antihypertensive potential of different varieties of tamarinds, and the sour type of tamarinds displayed the best effect in an animal model. Nonetheless, all tamarind varieties may be useful and beneficial as supplements or nutraceuticals for hypertensive patients. Future studies may approach the therapeutic effect of tamarind on hypertension through further extensive and target-based research. Molecular docking, network pharmacological analysis, and molecular dynamic simulation validated the study with consistent evidence, while pure isolated compounds from tamarinds would have been more impactful to affirm the biological potential of tamarinds in hypertension via elucidating the pure target ligand–receptor interactions.

## Figures and Tables

**Table 1:** Phytochemical screening of *Tamarindus indica*.

Tests	Biochemical Test	Observation	Result
Carbohydrates	Fehling’s test	Brick-red PPT	+
Proteins	Biuret test (Piotrowski’s test)	Purple color	+
Alkaloids	Wagner’s test	Reddish-brown PPT	+
Glycosides	Keller–Kiliani test	No color formation	-
Tannins	Lead acetate test	White PPT	+
Phenols	Lead acetate test	White PPT	+
Saponins	Froth test	Froth formation	+
Steroids	Salkowski’s test	Red color	+
Terpenoids	Salkowski’s test	Reddish-brown color	+
Flavonoids	Alkaline reagent test	Light yellow color	+
Sterols			-

PPT = Precipitate formation, froth = presence of froth in the test tube. (+) = present, (-) = absent.

**Table 2:** Total flavonoid content (TFC), total phenolic content (TPC), total proanthocyanidin content (TPrAC), and total antioxidant capacity (TAC) of different parts of *Tamarindus indica* fruit aqueous extract (TIFAE).

Parts of TIFAE	TFC, RE (mg/g)	TPC, GAE (mg/g)	TPrAC, Catechin (mg/g)	TAC, Catechin (mg/g)
FRiST	95.33 ± 1.39 ^a^	185.81 ± 0.55 ^a^	26.63 ± 0.09 ^a^	62.91 ± 2.46 ^a^
SRiST	173.76 ± 0.74 ^b^	63.54 ± 0.22 ^b^	84.41 ± 4.98 ^b^	56.66 ± 1.46 ^b^
FRaST	133.50 ± 1.17 ^c^	236.16 ± 0.60 ^c^	153.86 ± 1.97 ^c^	62.91 ± 0.97 ^a^
FSwT	115.47 ± 0.56 ^d^	240.94 ± 0.52 ^c^	45.52 ± 1.48 ^d^	181.63 ± 1.78 ^c^

Each value in the table is represented as mean ± SD for *n* = 3; FRiST = flesh of ripened sour tamarind; SRiST = seeds of ripened sour tamarind; FRaST = flesh of raw sour tamarind; FSwT = flesh of sweet tamarind; TFC = milligrams of rutin equivalent (RE) per gram of dried fruit/seed; TPC = milligrams of gallic acid equivalent (GAE) per milligrams of dried fruit/seed; TPrAC = milligrams of catechin equivalent per g of dried fruit/seed; TAC = milligrams of catechin equivalents per gram of dried fruit/seed. The superscript letters ^a–d^ in the columns illustrate the significant differences (*p* < 0.05) among the groups in the experimental conditions.

**Table 3:** Effects of different parts of *Tamarindus indica* fruit aqueous extracts on body weight gain in the experimental groups.

Groups	Week 1 (g)	(%) of Change	Week 2 (g)	(%) of Change
Normal Control	209.60 ± 1.91	-	210.64 ± 2.14	1.64 ± 0.87
Hypertensive Control	244.61 ± 3.24 ^a^***	16.72 ± 2.48	265.15 ± 3.58 ^a^***	26.51 ± 1.92
Reference Control	221.69 ± 2.82 ^b^***	5.78 ± 1.58	228.15 ± 2.76 ^b^***	8.86 ± 1.48
FRiST 50	229.63 ± 1.93 ^b^***	9.56 ± 1.20	232.96 ± 2.60 ^b^***	11.15 ± 1.57
SRiST 50	236.54 ± 1.12 ^b^*	12.86 ± 0.63	248.43 ± 2.59 ^b^***	18.53 ± 0.54
FRaST 50	241.12 ± 2.34	15.05 ± 1.92	246.93 ± 2.18 ^b^**	17.83 ± 1.91
FSwT 50	234.74 ± 1.62 ^b^*	12.00 ± 0.46	243.13 ± 2.16 ^b^***	16.00 ± 0.48
FRiST 100	230.61 ± 4.94 ^b^*	10.04 ± 2.78	238.94 ± 4.77 ^b^***	14.00 ± 2.26
SRiST 100	237.57 ± 2.07 ^b^*	13.35 ± 1.18	251.63 ± 3.92 ^b^**	20.05 ± 1.38
FRaST 100	243.08 ± 2.19 ^b^*	15.99 ± 1.88	256.33 ± 3.40 ^b^*	22.31 ± 2.52
FSwT 100	235.68 ± 3.48 ^b^*	12.45 ± 1.55	246.13 ± 2.50 ^b^***	17.43 ± 0.92

Each value represents the mean ± SD for *n* = 5 and was analyzed by one-way ANOVA, followed by *t*-tests. FRiST 50 and FRiST 100 = flesh of ripened sour tamarind aqueous extract at 50 g/kg BW and 100 mg/kg BW; SRiST 50 and SRiST 100 = seeds of ripened sour tamarind aqueous extract at 50 g/kg BW and 100 mg/kg BW; FRaST 50 and FRaST 100 = flesh of raw sour tamarind aqueous extract at 50 mg/kg BW and 100 mg/kg BW; FSwT 50 and FSwT 100 = flesh of sweet tamarind aqueous extract at 50 mg/kg BW and 100 mg/kg BW. Normal control vs. hypertensive control: *p* < 0.001 = a***; hypertensive control vs. treatment control: *p* < 0.05 = b*; *p* < 0.01 = b**; *p* < 0.001 = b***. Normal control group = non-hypertensive animals; hypertensive control group = developed hypertension and left untreated; reference control group = developed hypertension and treated with a reference drug.

**Table 4:** Effects of tamarind products on serum lipid profiles of experimental groups.

Groups	Cholesterol (mg/dL)	Triglycerides (mg/dL)	LDL (mg/dL)	HDL (mg/dL)	VLDL (mg/dL)
Normal Control	38.17 ± 4.17	51.57 ± 2.59	6.76 ± 0.39	47.14 ± 0.57	14.91 ± 0.76
Hypertensive Control	81.59 ± 5.13 ^a^***	86.38 ± 2.49 ^a^***	33.56 ± 3.10 ^a^***	31.24 ± 0.68 ^a^**	21.27 ± 0.61 ^a^***
Reference Control	58.19 ± 6.18 ^b^**	55.16 ± 2.65 ^b^***	15.77 ± 1.55 ^b^***	48.42 ± 0.74 ^b^***	17.73 ± 0.55 ^b^***
FRiST 50	39.64 ± 6.71 ^b^**	75.87 ± 1.56 ^b^**	8.22 ± 0.36 ^b^***	50.48 ± 0.80 ^b^***	13.92 ± 0.56 ^b^***
FRiST 100	46.12 ± 5.30 ^b^***	73.35 ± 3.27 ^b^***	10.53 ± 1.14 ^b^***	51.28 ± 0.68 ^b^***	18.63 ± 1.45 ^b^*
SRiST 50	45.58 ± 5.72 ^b^**	62.11 ± 2.97 ^b^***	8.68 ± 0.30 ^b^***	46.60 ± 0.72 ^b^***	11.34 ± 0.71 ^b^***
SRiST 100	41.23 ± 4.17 ^b^***	62.19 ± 5.75 ^b^**	9.22 ± 0.71 b***	50.42 ± 0.88 b***	10.73 ± 0.53 ^b^***
FRaST 50	52.43 ± 9.60 ^b^**	58.84 ± 3.71 ^b^***	9.01 ± 0.41 ^b^***	44.33 ± 0.80 ^b^***	10.79 ± 0.89 ^b^***
FRaST 100	65.51 ± 6.22 ^b^***	95.48 ± 15.30	10.72 ± 1.52 ^b^***	51.50 ± 0.94 ^b^***	21.44 ± 2.47
FSwT 50	68.34 ± 3.28 ^b^*	62.28 ± 4.64 ^b^***	11.89 ± 1.27 ^b^***	49.27 ± 2.16 ^b^***	12.37 ± 0.93 ^b^***
FSwT 100	42.83 ± 9.40 ^b^**	59.75 ± 4.53 ^b^***	6.43 ± 0.46 ^b^***	52.47 ± 2.24 ^b^***	15.37 ± 0.70 ^b^***

Each value represents the mean ± SD for *n* = 5 as analyzed by one-way ANOVA followed by *t*-tests. FRiST 50 and FRiST 100 = flesh of ripened sour tamarind aqueous extract at 50 mg/kg BW and 100 mg/kg BW; SRiST 50 and SRiST 100 = seeds of ripened sour tamarind aqueous extract at 50 mg/kg BW and 100 mg/kg BW; FRaST 50 and FRaST 100 = flesh of raw sour tamarind aqueous extract at 50 mg/kg BW and 100 mg/kg BW; FSwT 50 and FSwT 100 = flesh of sweet tamarind aqueous extract at 50 mg/kg BW and 100 mg/kg BW. Normal control vs. hypertensive control: *p* < 0.001 = a***; hypertensive control vs. treatment controls: *p* < 0.05 = b*; *p* < 0.01 = b**; *p* < 0.001 = b***. Normal control group = non-hypertensive animals; hypertensive control group = developed hypertension and left untreated; reference control group = developed hypertension and treated with a reference drug.

**Table 5:** Effects of tamarind products on serum enzymes of experimental groups.

Groups	ALT (IU/L)	AST (IU/L)	ALP (IU/L)
Normal Control	56.26 + 1.73	48.29 + 8.35	361.33 + 4.36
Hypertensive Control	297.59 + 12.06 ^a^***	166.77 + 5.78 ^a^***	582.93 + 8.06 ^a^***
Reference Control	128.94 + 4.65 ^b^***	60.06 + 3.24 ^b^***	466.73 + 4.13 ^b^***
FRiST50	120.44 + 5.01 ^b^***	54.98 + 3.44 ^b^***	451.53 + 6.31 ^b^***
FRiST100	187.84 + 4.46 ^b^***	98.97 + 3.37 ^b^***	291.85 + 5.33 ^b^***
SRiST50	129.22 + 5.94 ^b^***	64.58 + 3.94 ^b^***	222.83 + 5.18 ^b^***
SRiST100	147.86 + 3.20 ^b^***	70.01 + 3.03 ^b^***	273.86 + 4.13 ^b^***
FRaST50	145.14 + 4.32 ^b^***	69.26 + 2.84 ^b^***	385.07 + 5.45 ^b^***
FRaST100	133.00 + 2.42 ^b^***	73.44 + 3.67 ^b^***	382.18 + 6.18 ^b^***
FSwT50	161.73 + 5.56 ^b^***	58.02 + 0.82 ^b^***	365.65 + 7.19 ^b^***
FSwT100	99.88 + 3.15 ^b^***	62.57 + 1.95 ^b^***	310.88 + 12.81 ^b^***

Each value represents the mean ± SD for *n* = 5 as analyzed by one-way ANOVA followed by *t*-tests. FRiST 50 and FRiST 100 = flesh of ripened sour tamarind aqueous extract at 50 mg/kg BW and 100 mg/kg BW; SRiST 50 and SRiST 100 = seeds of ripened sour tamarind aqueous extract at 50 mg/kg BW and 100 mg/kg BW; FRaST 50 and FRaST 100 = flesh of raw sour tamarind aqueous extract at 50 mg/kg BW and 100 mg/kg BW; FSwT 50 and FSwT 100= flesh of sweet tamarind aqueous extract at 50 mg/kg BW and 100 mg/kg BW. Normal control vs. hypertensive control: *p* < 0.001 = a***; hypertensive control vs. treatment controls: *p* < 0.001 = b***. Normal control group = non-hypertensive animals; hypertensive control group = developed hypertension and left untreated; reference control group = developed hypertension and treated with a reference drug.

**Table 6:** Gas chromatography-mass spectrometry analysis data for fresh and ripened tamarind.

		Sour Tamarind Flesh			Sweet Tamarind Flesh
RT	Abd	Compounds	RT	Abd	Compounds
3.558	0.22	Dodecanoic acid, 3-hydroxy-	3.573	0.29	n-PROPYL NONYL ETHER
3.631	0.36	N-Glycylglycine	3.646	0.39	dl-2-Aminocaprylic acid
3.763	0.87	2,5,5-Trimethyl-3-hexyn-2-ol	3.733	0.29	Pentanoic acid, 5-(1-oxo-2-phenylethylamino)
3.850	0.26	3-Furancarboxylic acid	3.793	0.54	2,5-Dimethylfuran-3,4(2H,5H)-dione
4.001	1.46	Cyclohexanamine, N-3-butenyl-N-methyl-	4.025	1.96	Thymine
4.087	1.91	Methyl 2-furoate	4.094	0.58	Furyl hydroxymethyl ketone
4.208	0.47	2-Butenedioic acid (E)-, monomethyl ester	4.482	0.31	Aluminum, triethyl-
4.317	0.21	Hexadecanoic acid, 1,1-dimethylethyl ester	4.643	0.28	Ethanamine, N-ethyl-N-nitroso-
4.393	0.40	Levoglucosenone	4.766	2.30	4H-Pyran-4-one, 2,3-dihydro-3,5-dihydroxy-6
4.477	0.59	Heptane, 4-ethyl-	4.892	0.32	N,N-Dimethyl-O-(1-methyl-butyl)-hydroxylam
4.635	0.49	Glutaric acid, 3-heptyl propyl ester	4.942	0.17	2(3H)-Furanone, dihydro-4-hydroxy-
4.748	2.10	H-Pyran-4-one, 2,3-dihydro-3,5-dihydroxy-6	5.173	0.56	1,3,2-Dioxaborolan-4-one, 2-ethyl-5-methyl-
4.783	2.31	1-(2-Thienyl)-1-propanone	5.333	0.57	Pentanoic acid, 2-isopropoxyphenyl ester
4.983	0.01	2-Furanethanol, .beta.-methoxy-(S)-	5.457	0.16	Piperidine, 1-nitroso-
5.058	0.03	2(3H)-Furanone, dihydro-4-hydroxy-	5.722	12.74	1-Ethyl-2-hydroxymethylimidazole
5.167	0.06	4H-Pyran-4-one, 3,5-dihydroxy-2-methyl-	5.911	0.31	4-(Prop-2-enoyloxy)pentadecane
5.225	0.40	1,3-Propanediol, 2-(hydroxymethyl)-2-nitro-	6.292	0.27	Cyclobutanecarboxylic acid, heptyl ester
5.284	0.24	2-Propyl-1-pentanol	6.389	0.50	2-Methyl-1-di(tert-butyl)silyloxypropane
5.773	15.22	1-Ethyl-2-hydroxymethyl imidazole	6.595	0.59	3-cis-Methoxy-5-cis-methyl-1R-cyclohexanol
5.796	1.80	Fumaric acid, butyl 3-methylbut-3-enyl ester	6.776	0.48	2,4-Dimethyl-3-pentanol acetate
6.005	0.58	7-Dimethyl(prop-2-enyl)silyloxytridecane	7.202	0.38	Pentane, 2,2,4,4-tetramethyl-3-methoxy-
6.208	0.21	cis-13-Octadecenoic acid	7.449	2.12	7-Tridecanol
6.325	2.26	5-(Hydroxymethyl)-2-(dimethoxymethyl)furan	7.567	3.07	Succinic acid, di(but-2-en-1-yl) ester
6.449	1.30	Decanoic acid, 3-methyl-	7.791	0.55	Benzene, 1-chloro-4-methoxy-
6.640	0.86	2,4-Dimethyl-3-pentanol acetate	8.038	0.27	Galactopyranoside, 1-octylthio-1-deoxy-
6.811	0.54	Silane, dimethyldi(but-3-enyloxy)-	8.233	0.18	2-Acetyl-5-methylthiophene
7.213	0.22	.alpha.-Methyl mannofuranoside	8.362	1.05	Sucrose
7.538	2.36	Glutamine	8.558	0.18	Dinocap
7.636	0.43	3-Chloro-6-methoxy-2-methyl benzoic acid, m	9.225	0.25	1,6-Anhydro-.beta.-d-talopyranose
7.825	0.13	Carbonic acid, butyl ethyl ester	9.39	2.77	1,3-Propanediol, 2-(hydroxymethyl)-2-nitro-
7.958	0.07	(S)-(-)-1,2,4-Butanetriol, 4-acetate	10.618	0.28	.alpha.-Methyl-l-sorboside
8.036	0.19	1-Methyl-1-n-pentyloxy-1-silacyclobutane	10.753	1.34	1,6-Anhydro-.beta.-D-glucofuranose
8.367	0.25	D-Mannoheptulose	10.043	0.44	.alpha.-Methyl mannofuranoside
8.642	0.10	2-t-Butyl-4-oxo oxazolidine-3-carboxylic acid	12.408	16.53	D-Fructose, 3-O-methyl-
9.150	5.40	D-Allose	12.959	44.16	3-O-Methyl-d-glucose
9.281	3.89	.beta.-D-Glucopyranose, 1,6-anhydro-	27.663	0.98	0.98.gamma.-Sitosterol
9.779	0.20	1,2-O-Isopropylidene-D-xylofuranose, TBDM			
10.147	0.42	Acetic acid, 2-ethylbutyl ester			
10.317	0.13	3-Methylmannoside			
10.400	0.32	4-Hydroxy-3-[3-(2-hydroxy-5-methoxy-phenyl			
10.863	3.97	1,6-Anhydro-.beta.-D-glucofuranose			
11.226	0.50	Hydrazinecarboxamide, 2-(2-methylcyclohexy			
12.565	37.25	3-O-Methyl-d-glucose			
13.908	0.30	1-(2-Fluorophenyl)pyrazole-4-carboxylic acid			
14.908	0.49	4-Hydroxy-2-hydroxymethyl-6-methylpyrimidi			
17.958	0.29	R-(+)-Methyl-3-isopropyl-6-oxoheptanoate			
18.690	0.32	E-8-Methyl-7-dodecane-1-ol acetate			

**Table 7:** Toxicological properties of the selected compounds in *Tamarindus indica*.

Compounds	Parameters			
	Ames Toxicity	Carcinogens	Acute Oral Toxicity	Rat Acute Toxicity
(1-Ethyl-1H-imidazole-2-yl)methanol	NAT	NC	III	2.2439
4H-Pyran-4-one	AT	NC	III	1.7885
2-Furanmethanol,5-(dimethoxymethyl)-	NAT	NC	III	2.0967
7-Tridecanol	NAT	C	III	1.7615
D-Mannoheptulose	NAT	NC	IV	1.4430
Gamma-sitosterol	NAT	NC	I	2.6561
Glutamine	NAT	NC	IV	1.2587
1-(2-Thienyl)-1-propanone	NAT	NC	III	2.1210
Succinic acid, di(but-2-en-1-yl) ester	NAT	NC	III	1.8106
2-(Hydroxymethyl)-2-nitro-1,3-propanediol	NAT	NC	III	2.0756

NAT = non-Ames toxic; AT = Ames toxic; C = carcinogenic; NC = non-carcinogenic. Category I: ≤50 mg/kg; category III: 50 mg/kg < LD50 < 300 mg/kg; category IV: 300 mg/kg < LD50 < 2000 mg/kg.

**Table 8:** The binding affinity of most bioactive compounds (gamma-sitosterol) with different proteins.

	Binding Affinity
Protein Name	Gamma-Sitosterol (Compound ID: 457801)
Tyrosine hydroxylase (PDB ID: 1TOH)	−7.6
BETA-1 subunit of the soluble guanylyl cyclase (PDB ID: 3HLS)	−7.6
Human high-conductance Ca2+-gated K+ channel (BK Channel) (PDB ID: 3NAF)	−7.7
Nuclear hormone receptor PPAR-gamma (PDB ID: 3R8A)	−7.6
Human angiotensin receptor (PDB ID: 4YAY)	−9.3
Thermolysin (PDB ID: 5DPF)	−9.6
Macrocyclic IL-17A antagonists (PDB ID: 5HI3)	−9.1
Human soluble guanylate cyclase (PDB ID: 6JT0)	−7

Out of the selected compounds, only 7-tridecanol was found to show carcinogenicity. 7-Tridecanol is carcinogenic to aquatic organisms, but insufficient data are available on the effect of this substance on human health. The compounds’ oral toxicity levels were mostly III, showing an LD50 between 50 mg/kg and 300 mg/kg, except for D-Mannoheptulose and glutamine, which had an LD50 between 300 mg/kg and 2000 mg/kg.

**Table 9:** Pharmacokinetic properties of the selected compounds in different *T. indica* extracts.

Tamarind Compounds	Lipinski Rules				Lipinski’s Violations (≤1)	Veber Rules	
	MW (<500)	HBA (<10)	HBD (<5)	Log P (≤5)		Nrb (≤10)	TPSA (≤140 Å^2^)
(1-Ethyl-1H-imidazol-2-yl)methanol	126.16	2	1	0.21	0	2	38.05
4H-Pyran-4-one	144.13	4	2	−0.22	0	0	66.76
2-Furanmethanol,5-(dimethoxymethyl)-	172.18	4	1	0.60	0	4	51.83
7-Tridecanol	200.36	1	1	4.28	0	10	20.23
D-Mannoheptulose	210.18	7	6	−2.65	1	7	107.22
Gamma-sitosterol	414.71	1	1	7.19	1	6	20.23
Glutamine	146.14	4	3	−1.81	0	4	106.41
1-(2-Thienyl)-1-propanone	140.20	1	0	2.03	0	2	45.31
Succinic acid, di(but-2-en-1-yl) ester	226.27	4	0	2.26	0	9	52.60
2-(Hydroxymethyl)-2-nitro-1,3-propanediol	151.12	5	3	−1.84	0	4	106.51

MW = molecular weight; HBA = hydrogen bond acceptor; HBD = hydrogen bond donor; Log P = liphophilicity.

## Data Availability

All data are included in this manuscript. Raw data may be available upon request.

## Supplementary Materials

The following supporting information can be downloaded at: https://www.mdpi.com/article/10.3390/nu15153402/s1, Table S1: Hypertension related Genes (HRG) and Compound targeted Genes (CTG); Table S2: Genes related to Hypertension. Source: GeneCard; Table S3: Gene Ontology Enrichment (Biological Process); Table S4: Gene Ontology Enrichment (Molecular Function); Table S5: Gene Ontology Enrichment (Cellular Component); Table S6: KEGG Pathways. Video S1: Biological stability of the best binding ligand with receptor protein.

## Abbreviations

CRP	C-reactive protein
WBC	White blood cell
LDL	Lactate dehydrogenase
HDL	High-density lipoprotein
NC	Normal control group
PC	Positive control group
TC	Total cholesterol
TG	Triglyceride
LDL	Low-density lipoprotein
VLDL	Very low-density lipoprotein
AST	Serum aspartate aminotransferase
ALT	Alanine aminotransferase
ALP	Alkaline phosphatase
GAE	Gallic acid equivalents
HTN	Hypertension
TPC	Total phenolic content
TFC	Total flavonoid content
FRiST	Flesh of ripened sour tamarind
SRiST	Seeds of ripened sour tamarind
FRaST	Flesh of raw sour tamarind
FSwT	Flesh of sweet tamarind
