# Supplementary material for: Sour Tamarind Is More Antihypertensive than the Sweeter One, as Evidenced by In Vivo Biochemical Indexes, Ligand–Protein Interactions, Multitarget Interactions, and Molecular Dynamic Simulation
Source: Nutrients. 2023 Jul 31;15(15):3402. doi: 10.3390/nu15153402 (PMC10420995; doi:10.3390/nu15153402)
Supplement: Supplementary file 1 [file nutrients-15-03402-s001.zip › nutrients-2472603-supplementary tables.pdf]

Supplementary Materials:

Table S1: Hypertension related Genes (HRG) and Compound targeted Genes (CTG).

Names	total	elements
CTG HRG	14	CYP17A1 NR3C2 KDR NOS2 PTPN1 JAK2 PTGS2 REN HSD11B1 CYP11B2 PPARG HIF1A CYP11B1 NR3C1
HRG	228	ACE ABCA3 MMP2 ECE1 LEPR MTOR BMP6 RGS2 NPPA EDN1 DIPK1A COL3A1 ENG SERPINE1 STOX1 CACNA1D CYBA IGF1 MIR20A MIR17 VCAM1 RGS5 CYP3A5 CCN2 TP53 PIEZO2 LOC106799833 ADRB1 HMOX1 NKX2-5 TH INS TNF FLT1 INSR MEN1 COL4A5 GDF2 STN1 LOX SMAD2 NEDD4L HNF1B KCNK3 UMOD CACNA1H HYT1 TBX4 CCR6 SDHB LPL HLA-B LOC102723566 SGK1 ELN SELP LEP KCNMB1 GJA1 SLC12A3 PKD1 F5 ENPP1 SCNN1A PRKAR1A HSD11B2 CPS1 EDNRB ACVRL1 BMPR2 ACSM3 GSTM1 NOS1 IL10 PKD2 BMPR1A ADRB3 PEE3 ADM PGF SPP1 TEK HYT6 SARS2 F2 DGUOK GIMAP5 MTHFR PRKG1 SMAD3 PEE2 SDHD B2M NF1 EDN2 OLR1 PLAT NPR3 GNAS NCF1 COL4A4 CBS CTNNB1 UTS2 AKT2 EGFR SOX17 MIR204 NPPB RPL5 HYT2 IL1B VWF MYLK SOD1 WNK4 NPY HYT5 RBPJ ADD1 APOE POMC ALDH2 ATP1B1 C3 HYT3 F12 RFH1 SST FGA PON1 ACE2 AGTR1 MIR759 ACTA2 SCNN1B ALB RETN SLC12A1 SERPINC1 F3 CUL3 MYH6 HTR2A SCNN1G KCNJ5 LMNA ATP13A3 CTEPH1 APOA1 CD36 PDE3A ENPEP GIMAP1-GIMAP5 ADRB2 BDKRB2 SMAD4 HYT8 VEGFA WNK1 PDE5A TGFB1 KCNJ11 EDNRA ABCC8 MTX2 IRS1 ICAM1 CAV1 NOTCH3 SMAD9 FBN1 GBA1 TIMP1 APOB CFH NFU1 CORIN FOXF1 AGTR2 AGT KLHL3 EPO HYT4 MIR21 APLN TRPC6 NOTCH1 CST3 CHGA PKHD1 SH2B3 GPT CRP CCL2 CLCNKB CALCA SLC2A4 MIR155 ADD2 CELA2A MIR29A RET NOS3 IL6 PEE1 ANGPT1 HTNB GATA4 KCNA5 VHL COMT MIR122 ENSG00000230926 PTGIS KNG1 SELE FN1 GRK4 GNB3 HYT7 EIF2AK4 PMS2 SLC34A1 MMP9 THBD DARS2 ADIPOQ
CTG	201	VDR PPARD HMGCR NPY5R PRKCE GABBR1 PRCP CHRM4 SLC6A2 GSR TK1 GRM7 CYP19A1 MAPK14 HSD17B2 AOC3 FABP3 IDO1 GFPT1 GRM3 CTSS PRKCG LCK CACNA2D1 SLC6A4 GRM6 FNTA MMP3 CHRM5 SERPINA6 CRHR1 PDE2A CDK5 PPARA GRM2 CNR2 RORA SLC1A2 CDK9 PRKCQ OGA ALOX5 JAK3 SLC6A12 APH1B JAK1 CCR1 MAOB PTGER2 BACE1 TRPM8 KIT GLRA1 FNTB FABP4 SHH CDC25B S1PR2 PRKCH PRKCB GRM8 CYP2C19 NR1H2 NPY2R CA4 STAT3 NR1H3 SLC6A9 PYGL ELANE GRIK1 PYGB UGT2B7 CCR9 GRIA1 RORC TRPA1 FDFT1 HDAC1 SQLE PSENEN GRM4 CES2 NPC1L1 MIF TYK2 CYP2C9 CHRM3 NCSTN CCNE1 GABBR2 GCGR CTSK BRS3 GPBAR1 MAOA TSPO OPRL1 ADORA1 KDM4C GRIK5 ACLY ESR1 SLC1A1 AKR1C3 NAAA AGPAT2 TYMS SHBG FABP5 APH1A PTGS1 GRIA4 GRM1 BCHE NR1I2 EPAS1 GRM5 TTL PYGM PER2 HAO1 PSEN1 CCNT1 DHCR7 CTSD SREBF2 G6PD GCK DAO ADORA2A MAPK8 AVPR1A KCNH2 PSEN2 LIPE F10 CA1 F2R CSF1R AVPR2 ADORA3 DHODH GRIK2 FABP2 CDA LRRK2 OXTR ADK MAPK10 AR SLC10A2 CA2 IL6ST ESR2 PTGES FFAR1 CSNK2A1 ATP6V1B1 ACHE CHRM1 GSK3B ALOX15 CDK5R1 TERT FUCA1 PRKCA PRKCD CES1 ADA AKR1C2 MAP2 CYP3A4 GPR88 CYP51A1 POLB EPHX2 SCN9A GRIA2 PTPN6 APP MAPK9 PTPN2 GRIK3 PDE10A PTGFR PTGER1 CDK2 CNR1 TRPV1 PGR CHRM2 CHRNA7 NR1H4 ODC1 ADORA2B NR1I3 PGA5 CDC25A KDM4E OPRM1

Table S2: Genes related to Hypertension. Source: GeneCard

Gene Symbol	Description	Category	Gifts	GC Id	Relevance score
BMPR2	Bone Morphogenetic Protein Receptor Type 2	Protein Coding	57	GC02P202376	90.31098175
AGTR1	Angiotensin II Receptor Type 1	Protein Coding	58	GC03P148697	54.02256012
AGT	Angiotensinogen	Protein Coding	56	GC01M230690	53.16813278
NOS3	Nitric Oxide Synthase 3	Protein Coding	56	GC07P151271	52.31625748
ACVRL1	Activin A Receptor Like Type 1	Protein Coding	57	GC12P051906	46.75440216
ACE	Angiotensin I Converting Enzyme	Protein Coding	59	GC17P063477	46.07898712
TBX4	T-Box Transcription Factor 4	Protein Coding	47	GC17P067536	44.997715
KCNK3	Potassium Two Pore Domain Channel Subfamily K Member 3	Protein Coding	57	GC02P026692	44.67465591
ENG	Endoglin	Protein Coding	55	GC09M127815	42.46354675
GNB3	G Protein Subunit Beta 3	Protein Coding	53	GC12P006839	41.56012726
ADD1	Adducin 1	Protein Coding	51	GC04P002859	38.75555038
NR3C2	Nuclear Receptor Subfamily 3 Group C Member 2	Protein Coding	52	GC04M148078	37.97737122
HYT4	Hypertension, Essential, Susceptibility To, 4	Genetic Locus	2	GC12U900475	34.7195816
HYT3	Hypertension, Essential, Susceptibility To, 3	Genetic Locus	2	GC02U900228	34.66175461
HYT8	Hypertension, Essential, Susceptibility To, 8	Genetic Locus	2	GC18U900482	34.63870621
HYT1	Hypertension, Essential, Susceptibility To, 1	Genetic Locus	2	GC17U990303	34.58152008
PKD1	Polycystin 1, Transient Receptor Potential Channel Interacting	Protein Coding	52	GC16M008593	34.48180008
HYT2	Hypertension, Essential, Susceptibility To, 2	Genetic Locus	2	GC15U990105	34.10437393
HYT5	Hypertension, Essential, Susceptibility To, 5	Genetic Locus	2	GC20U900425	33.95214081
HYT6	Hypertension, Essential, Susceptibility To, 6	Genetic Locus	2	GC05U901045	33.95214081
HYT7	Hypertension, Essential, Susceptibility To, 7	Genetic Locus	2	GC03U901240	33.79850769
ATP13A3	ATPase 13A3	Protein Coding	46	GC03M194402	32.51779175
CYP11B2	Cytochrome P450 Family 11 Subfamily B Member 2	Protein Coding	53	GC08M142910	32.16856003
EIF2AK4	Eukaryotic Translation Initiation Factor 2 Alpha Kinase 4	Protein Coding	52	GC15P039934	31.96027374
REN	Renin	Protein Coding	55	GC01M204154	31.79884911
PTGIS	Prostaglandin I2 Synthase	Protein Coding	52	GC20M049503	31.71622467
ECE1	Endothelin Converting Enzyme 1	Protein Coding	55	GC01M021217	31.27714157
CAV1	Caveolin 1	Protein Coding	55	GC07P116524	30.97431183

PPARG	Peroxisome Proliferator Activated Receptor Gamma	Protein Coding	60	GC03P012287	30.9062252
PDE3A	Phosphodiesterase 3A	Protein Coding	56	GC12P025362	30.37831497
PKD2	Polycystin 2, Transient Receptor Potential Cation Channel	Protein Coding	54	GC04P088007	29.99750519
MEN1	Menin 1	Protein Coding	51	GC11M064803	29.29608727
SMAD9	SMAD Family Member 9	Protein Coding	53	GC13M036844	28.87478828
CYP3A5	Cytochrome P450 Family 3 Subfamily A Member 5	Protein Coding	51	GC07M099648	28.72484398
COL4A5	Collagen Type IV Alpha 5 Chain	Protein Coding	51	GC0XP108439	28.33776283
KLHL3	Kelch Like Family Member 3	Protein Coding	47	GC05M137617	27.98581696
CELA2A	Chymotrypsin Like Elastase 2A	Protein Coding	48	GC01P015456	27.79840279
MIR155	MicroRNA 155	RNA Gene	23	GC21P025573	26.6363163
CYP11B1	Cytochrome P450 Family 11 Subfamily B Member 1	Protein Coding	52	GC08M142872	25.95476913
EDN1	Endothelin 1	Protein Coding	55	GC06P012256	25.75426292
F12	Coagulation Factor XII	Protein Coding	56	GC05M177402	25.18717003
LOC102723566	Uncharacterized LOC102723566	RNA Gene	15	GC09P127816	24.93225479
CORIN	Corin, Serine Peptidase	Protein Coding	51	GC04M047596	24.92873573
FLT1	Fms Related Receptor Tyrosine Kinase 1	Protein Coding	57	GC13M028300	24.87867355
SARS2	Seryl-TRNA Synthetase 2, Mitochondrial	Protein Coding	51	GC19M073555	24.83227348
DARS2	Aspartyl-TRNA Synthetase 2, Mitochondrial	Protein Coding	48	GC01P173824	24.59682083
HSD11B2	Hydroxysteroid 11-Beta Dehydrogenase 2	Protein Coding	52	GC16P067433	24.5582428
RGS5	Regulator Of G Protein Signaling 5	Protein Coding	47	GC01M163111	24.3645916
MTX2	Metaxin 2	Protein Coding	48	GC02P176269	24.21414757
SLC34A1	Solute Carrier Family 34 Member 1	Protein Coding	51	GC05P178655	24.17390251
ATP1B1	ATPase Na+/K+ Transporting Subunit Beta 1	Protein Coding	55	GC01P169105	24.10710526
SOX17	SRY-Box Transcription Factor 17	Protein Coding	49	GC08P054457	23.49224091
MIR204	MicroRNA 204	RNA Gene	26	GC09M070809	23.43336487
INS	Insulin	Protein Coding	54	GC11M002159	23.40236664
NPPA	Natriuretic Peptide A	Protein Coding	52	GC01M011846	23.35327911
CUL3	Cullin 3	Protein Coding	54	GC02M224470	22.70981598
IL6	Interleukin 6	Protein Coding	58	GC07P022725	22.36800575
ALB	Albumin	Protein Coding	56	GC04P073397	21.65635681

SCNN1B	Sodium Channel Epithelial 1 Subunit Beta	Protein Coding	55	GC16P023278	21.47747231
RFH1	Renal Failure, Progressive, With Hypertension	Genetic Locus	2	GC01U990346	20.68140793
MIR17	MicroRNA 17	RNA Gene	22	GC13P091350	20.51968956
PMS2	PMS1 Homolog 2, Mismatch Repair System Component	Protein Coding	55	GC07M005973	20.22091103
RPL5	Ribosomal Protein L5	Protein Coding	54	GC01P092832	19.85341454
KCNMB1	Potassium Calcium-Activated Channel Subfamily M Regulatory Beta Subunit 1	Protein Coding	50	GC05M170374	19.62186432
GIMAP5	GTPase, IMAP Family Member 5	Protein Coding	44	GC07P150722	19.55440903
NPPB	Natriuretic Peptide B	Protein Coding	50	GC01M011858	19.29896736
FOXF1	Forkhead Box F1	Protein Coding	48	GC16P086510	19.23005295
DGUOK	Deoxyguanosine Kinase	Protein Coding	51	GC02P073926	19.13512611
DIPK1A	Divergent Protein Kinase Domain 1A	Protein Coding	39	GC01M092833	19.11083794
CRP	C-Reactive Protein	Protein Coding	53	GC01M159730	18.93082809
KCNJ5	Potassium Inwardly Rectifying Channel Subfamily J Member 5	Protein Coding	53	GC11P128891	18.73002815
SCNN1G	Sodium Channel Epithelial 1 Subunit Gamma	Protein Coding	54	GC16P023182	18.65215874
WNK1	WNK Lysine Deficient Protein Kinase 1	Protein Coding	55	GC12P000733	18.5628624
MTHFR	Methylenetetrahydrofolate Reductase	Protein Coding	54	GC01M011785	18.20135689
PEE1	Preeclampsia/Eclampsia 1	Genetic Locus	3	GC04U902284	18.08944893
SERPINE1	Serpin Family E Member 1	Protein Coding	57	GC07P101127	17.89898109
ADRB2	Adrenoceptor Beta 2	Protein Coding	56	GC05P148825	17.82954979
WNK4	WNK Lysine Deficient Protein Kinase 4	Protein Coding	52	GC17P042780	17.72973061
SCNN1A	Sodium Channel Epithelial 1 Subunit Alpha	Protein Coding	55	GC12M006346	17.61868668
NR3C1	Nuclear Receptor Subfamily 3 Group C Member 1	Protein Coding	56	GC05M143277	17.55585861
TNF	Tumor Necrosis Factor	Protein Coding	59	GC06P096100	17.34045982
ADIPOQ	Adiponectin, C1Q And Collagen Domain Containing	Protein Coding	52	GC03P186842	16.73889351
CTEPH1	Pulmonary Hypertension, Chronic Thromboembolic, Without Deep Vein Thrombosis, Susceptibility To	Genetic Locus	2	GC06U901832	16.72580147
CPS1	Carbamoyl-Phosphate Synthase 1	Protein Coding	55	GC02P210477	16.59735298
CACNA1H	Calcium Voltage-Gated Channel Subunit Alpha1 H	Protein Coding	57	GC16P001153	16.48690033
INSR	Insulin Receptor	Protein Coding	60	GC19M007112	16.3680954

ELN	Elastin	Protein Coding	50	GC07P074027	16.36404419
CYP17A1	Cytochrome P450 Family 17 Subfamily A Member 1	Protein Coding	55	GC10M102830	16.33756447
STOX1	Storkhead Box 1	Protein Coding	46	GC10P068827	16.32596588
GNAS	GNAS Complex Locus	Protein Coding	56	GC20P058839	16.31542015
APOA1	Apolipoprotein A1	Protein Coding	57	GC11M116835	16.31117439
APOB	Apolipoprotein B	Protein Coding	53	GC02M020956	16.25160789
SDHB	Succinate Dehydrogenase Complex Iron Sulfur Subunit B	Protein Coding	54	GC01M018419	16.24979782
JAK2	Janus Kinase 2	Protein Coding	60	GC09P004985	16.1608429
APOE	Apolipoprotein E	Protein Coding	57	GC19P076400	16.08358955
ENPP1	Ectonucleotide Pyrophosphatase/Phosphodiesterase 1	Protein Coding	57	GC06P131808	15.87401676
RET	Ret Proto-Oncogene	Protein Coding	61	GC10P043271	15.83736897
RETN	Resistin	Protein Coding	49	GC19P007669	15.79308319
LOC106799833	CYP11B1 Recombination Region	Functional Element	4	GC08P142874	15.77501106
VEGFA	Vascular Endothelial Growth Factor A	Protein Coding	56	GC06P043770	15.73114395
FN1	Fibronectin 1	Protein Coding	57	GC02M215360	15.72671223
LEP	Leptin	Protein Coding	53	GC07P128241	15.61670876
THBD	Thrombomodulin	Protein Coding	53	GC20M023026	15.59465504
IRS1	Insulin Receptor Substrate 1	Protein Coding	53	GC02M226731	15.46720314
MIR20A	MicroRNA 20a	RNA Gene	22	GC13P091595	15.40781403
ADM	Adrenomedullin	Protein Coding	51	GC11P010304	15.06311226
FBN1	Fibrillin 1	Protein Coding	53	GC15M048408	15.04684639
F5	Coagulation Factor V	Protein Coding	53	GC01M169511	14.80826473
TGFB1	Transforming Growth Factor Beta 1	Protein Coding	60	GC19M041301	14.7720089
SMAD4	SMAD Family Member 4	Protein Coding	59	GC18P051028	14.75741959
F2	Coagulation Factor II, Thrombin	Protein Coding	56	GC11P046752	14.6315918
PIEZO2	Piezo Type Mechanosensitive Ion Channel Component 2	Protein Coding	43	GC18M010670	14.53896904
CACNA1D	Calcium Voltage-Gated Channel Subunit Alpha1 D	Protein Coding	54	GC03P053328	14.52237797
EDNRA	Endothelin Receptor Type A	Protein Coding	56	GC04P147480	14.45056152
VHL	Von Hippel-Lindau Tumor Suppressor	Protein Coding	54	GC03P013997	14.36838055

GDF2	Growth Differentiation Factor 2	Protein Coding	51	GC10P047322	14.31663132
GIMAP1-GIMAP5	GIMAP1-GIMAP5 Readthrough	Protein Coding	19	GC07P150716	14.2960186
KNG1	Kininogen 1	Protein Coding	55	GC03P186717	14.23037624
UMOD	Uromodulin	Protein Coding	49	GC16M020344	14.10329628
HMOX1	Heme Oxygenase 1	Protein Coding	59	GC22P035380	14.01594162
VWF	Von Willebrand Factor	Protein Coding	55	GC12M005949	14.01437473
MMP2	Matrix Metallopeptidase 2	Protein Coding	60	GC16P055390	13.95766068
BMPR1A	Bone Morphogenetic Protein Receptor Type 1A	Protein Coding	57	GC10P095535	13.90503311
SELP	Selectin P	Protein Coding	52	GC01M169558	13.84096432
PLAT	Plasminogen Activator, Tissue Type	Protein Coding	56	GC08M042174	13.64021015
AGTR2	Angiotensin II Receptor Type 2	Protein Coding	51	GC0XP116170	13.53675652
HLA-B	Major Histocompatibility Complex, Class I, B	Protein Coding	52	GC06M074887	13.53315353
TP53	Tumor Protein P53	Protein Coding	61	GC17M007661	13.39956856
ADRB1	Adrenoceptor Beta 1	Protein Coding	54	GC10P114044	13.39370918
NOS2	Nitric Oxide Synthase 2	Protein Coding	56	GC17M027756	13.26169491
ACE2	Angiotensin Converting Enzyme 2	Protein Coding	56	GC0XM015494	13.04372597
ADRB3	Adrenoceptor Beta 3	Protein Coding	51	GC08M037962	12.96000385
MIR759	MicroRNA 759	RNA Gene	16	GC13P052810	12.89013195
NOTCH1	Notch Receptor 1	Protein Coding	59	GC09M137930	12.88270855
PKHD1	PKHD1 Ciliary IPT Domain Containing Fibrocystin/Polyductin	Protein Coding	45	GC06M075258	12.86030293
CCN2	Cellular Communication Network Factor 2	Protein Coding	53	GC06M131948	12.81209564
MMP9	Matrix Metallopeptidase 9	Protein Coding	61	GC20P046008	12.65143585
SMAD3	SMAD Family Member 3	Protein Coding	59	GC15P067063	12.61476517
IGF1	Insulin Like Growth Factor 1	Protein Coding	55	GC12M102395	12.5779562
PEE2	Preeclampsia/Eclampsia 2	Genetic Locus	2	GC02U901135	12.57615566
PEE3	Preeclampsia/Eclampsia 3	Genetic Locus	2	GC09U900850	12.57615566
B2M	Beta-2-Microglobulin	Protein Coding	56	GC15P044711	12.4970293
LPL	Lipoprotein Lipase	Protein Coding	56	GC08P019901	12.47077751
ACTA2	Actin Alpha 2, Smooth Muscle	Protein Coding	53	GC10M088935	12.4609251

MIR29A	MicroRNA 29a	RNA Gene	24	GC07M130876	12.3916111
TEK	TEK Receptor Tyrosine Kinase	Protein Coding	58	GC09P027109	12.36257744
COMT	Catechol-O-Methyltransferase	Protein Coding	59	GC22P019941	12.3562603
SLC12A3	Solute Carrier Family 12 Member 3	Protein Coding	55	GC16P056865	12.23305321
CCL2	C-C Motif Chemokine Ligand 2	Protein Coding	56	GC17P034255	12.22761822
C3	Complement C3	Protein Coding	56	GC19M006677	12.16946316
COL4A4	Collagen Type IV Alpha 4 Chain	Protein Coding	51	GC02M226973	12.14629459
GATA4	GATA Binding Protein 4	Protein Coding	55	GC08P011676	12.13488293
SOD1	Superoxide Dismutase 1	Protein Coding	60	GC21P031659	12.11402988
SELE	Selectin E	Protein Coding	50	GC01M169722	12.10501862
EPO	Erythropoietin	Protein Coding	49	GC07P100720	12.0686636
PRKG1	Protein Kinase CGMP-Dependent 1	Protein Coding	57	GC10P050991	12.05807018
PTPN1	Protein Tyrosine Phosphatase Non-Receptor Type 1	Protein Coding	57	GC20P050510	12.0508194
SST	Somatostatin	Protein Coding	47	GC03M187668	11.99067497
CTNNB1	Catenin Beta 1	Protein Coding	60	GC03P041194	11.98919582
SLC2A4	Solute Carrier Family 2 Member 4	Protein Coding	52	GC17P012037	11.98107338
COL3A1	Collagen Type III Alpha 1 Chain	Protein Coding	54	GC02P188974	11.94369602
SMAD2	SMAD Family Member 2	Protein Coding	59	GC18M047809	11.86574078
PGF	Placental Growth Factor	Protein Coding	50	GC14M074941	11.82468033
NKX2-5	NK2 Homeobox 5	Protein Coding	51	GC05M173232	11.74453735
IL1B	Interleukin 1 Beta	Protein Coding	54	GC02M112829	11.72405243
UTS2	Urotensin 2	Protein Coding	46	GC01M007843	11.67801476
ABCC8	ATP Binding Cassette Subfamily C Member 8	Protein Coding	53	GC11M017392	11.65569592
CBS	Cystathionine Beta-Synthase	Protein Coding	56	GC21M043053	11.64024925
SGK1	Serum/Glucocorticoid Regulated Kinase 1	Protein Coding	56	GC06M134169	11.5962162
ICAM1	Intercellular Adhesion Molecule 1	Protein Coding	57	GC19P010870	11.55531311
FGA	Fibrinogen Alpha Chain	Protein Coding	56	GC04M154583	11.52323532
NPR3	Natriuretic Peptide Receptor 3	Protein Coding	52	GC05P032689	11.51934147
GRK4	G Protein-Coupled Receptor Kinase 4	Protein Coding	50	GC04P002963	11.50443935
PDE5A	Phosphodiesterase 5A	Protein Coding	52	GC04M119494	11.49082279

TH	Tyrosine Hydroxylase	Protein Coding	57	GC11M002163	11.48639679
CFH	Complement Factor H	Protein Coding	54	GC01P196621	11.46793938
HIF1A	Hypoxia Inducible Factor 1 Subunit Alpha	Protein Coding	54	GC14P061695	11.46062565
NF1	Neurofibromin 1	Protein Coding	54	GC17P031094	11.37448692
HNF1B	HNF1 Homeobox B	Protein Coding	50	GC17M037686	11.37330055
SH2B3	SH2B Adaptor Protein 3	Protein Coding	54	GC12P111405	11.33392429
GJA1	Gap Junction Protein Alpha 1	Protein Coding	57	GC06P121436	11.29053307
CLCNKB	Chloride Voltage-Gated Channel Kb	Protein Coding	50	GC01P016059	11.27181149
NCF1	Neutrophil Cytosolic Factor 1	Protein Coding	53	GC07P077733	11.27112389
KCNJ11	Potassium Inwardly Rectifying Channel Subfamily J Member 11	Protein Coding	53	GC11M017697	11.25201035
LOX	Lysyl Oxidase	Protein Coding	54	GC05M122063	11.204566
GBA1	Glucosylceramidase Beta 1	Protein Coding	56	GC01M155424	11.18928051
ENPEP	Glutamyl Aminopeptidase	Protein Coding	53	GC04P110365	11.16913319
CD36	CD36 Molecule	Protein Coding	56	GC07P080369	11.13616657
LMNA	Lamin A/C	Protein Coding	56	GC01P156082	11.11142254
BDKRB2	Bradykinin Receptor B2	Protein Coding	52	GC14P096205	11.10740948
F3	Coagulation Factor III, Tissue Factor	Protein Coding	52	GC01M094624	11.03021812
ALDH2	Aldehyde Dehydrogenase 2 Family Member	Protein Coding	57	GC12P111766	10.98056412
CALCA	Calcitonin Related Polypeptide Alpha	Protein Coding	50	GC11M014945	10.97874832
PON1	Paraoxonase 1	Protein Coding	55	GC07M095297	10.96961117
NEDD4L	NEDD4 Like E3 Ubiquitin Protein Ligase	Protein Coding	52	GC18P058044	10.96296978
HSD11B1	Hydroxysteroid 11-Beta Dehydrogenase 1	Protein Coding	58	GC01P209686	10.92437744
SDHD	Succinate Dehydrogenase Complex Subunit D	Protein Coding	51	GC11P112087	10.92064667
POMC	Proopiomelanocortin	Protein Coding	55	GC02M025160	10.89772987
MYH6	Myosin Heavy Chain 6	Protein Coding	52	GC14M023381	10.88994122
EGFR	Epidermal Growth Factor Receptor	Protein Coding	62	GC07P055019	10.83493233
TRPC6	Transient Receptor Potential Cation Channel Subfamily C Member 6	Protein Coding	57	GC11M101451	10.76990032
AKT2	AKT Serine/Threonine Kinase 2	Protein Coding	61	GC19M040230	10.69749451
TIMP1	TIMP Metallopeptidase Inhibitor 1	Protein Coding	52	GC0XP047769	10.69340897

CHGA	Chromogranin A	Protein Coding	50	GC14P092953	10.66917038
NFU1	NFU1 Iron-Sulfur Cluster Scaffold	Protein Coding	48	GC02M069395	10.66077042
CST3	Cystatin C	Protein Coding	51	GC20M023699	10.65707397
HTR2A	5-Hydroxytryptamine Receptor 2A	Protein Coding	55	GC13M046831	10.64847851
PRKAR1A	Protein Kinase CAMP-Dependent Type I Regulatory Subunit Alpha	Protein Coding	57	GC17P070537	10.61178684
EDNRB	Endothelin Receptor Type B	Protein Coding	56	GC13M077895	10.58321476
MIR122	MicroRNA 122	RNA Gene	25	GC18P058451	10.53453255
VCAM1	Vascular Cell Adhesion Molecule 1	Protein Coding	52	GC01P100719	10.52820873
CYBA	Cytochrome B-245 Alpha Chain	Protein Coding	54	GC16M088643	10.5258007
NPY	Neuropeptide Y	Protein Coding	51	GC07P024290	10.4707222
MIR21	MicroRNA 21	RNA Gene	26	GC17P067479	10.44579411
STN1	STN1 Subunit Of CST Complex	Protein Coding	46	GC10M103986	10.42180347
IL10	Interleukin 10	Protein Coding	55	GC01M206767	10.39445591
APLN	Apelin	Protein Coding	44	GC0XM129645	10.39334106
SLC12A1	Solute Carrier Family 12 Member 1	Protein Coding	54	GC15P049252	10.37599373
CCR6	C-C Motif Chemokine Receptor 6	Protein Coding	51	GC06P167111	10.36265469
SERPINC1	Serpin Family C Member 1	Protein Coding	56	GC01M174691	10.35053349
LEPR	Leptin Receptor	Protein Coding	57	GC01P065421	10.32778454
ENSG00000230926	Pseudogene Similar To Part Of SAH (SA Hypertension-Associated Homolog (Rat))	Pseudogene	2	GC0XP051663	10.3196888
HTNB	Hypertension With Brachydactyly	Genetic Locus	2	GC00U936608	10.3196888
ADD2	Adducin 2	Protein Coding	47	GC02M070626	10.3196125
GPT	Glutamic--Pyruvic Transaminase	Protein Coding	48	GC08P144502	10.31947041
RGS2	Regulator Of G Protein Signaling 2	Protein Coding	49	GC01P192809	10.27458668
RBPJ	Recombination Signal Binding Protein For Immunoglobulin Kappa J Region	Protein Coding	53	GC04P026105	10.25145149
KDR	Kinase Insert Domain Receptor	Protein Coding	60	GC04M055078	10.24802303
ABCA3	ATP Binding Cassette Subfamily A Member 3	Protein Coding	55	GC16M002275	10.23082829
ACSM3	Acyl-CoA Synthetase Medium Chain Family Member 3	Protein Coding	46	GC16P020610	10.20767021
SPP1	Secreted Phosphoprotein 1	Protein Coding	53	GC04P087975	10.18020058
BMP6	Bone Morphogenetic Protein 6	Protein Coding	51	GC06P007726	10.1758585
KCNA5	Potassium Voltage-Gated Channel Subfamily A Member 5	Protein Coding	52	GC12P005043	10.17279816

Table S3: Gene Ontology Enrichment (Biological Process)

Enrichment FDR	nGenes	Pathway Genes	Fold Enrichment	Pathway	Genes
6.93E-07	4	26	250.5054945	Glucocorticoid biosynthetic proc.	HSD11B1 CYP17A1 CYP11B1 CYP11B2
9.98E-07	4	32	203.5357143	Glucocorticoid metabolic proc.	HSD11B1 CYP17A1 CYP11B1 CYP11B2
2.12E-05	4	85	76.62521008	Hormone biosynthetic proc.	HIF1A CYP17A1 CYP11B1 CYP11B2
3.52E-05	4	103	63.23439667	Reg. of blood vessel endothelial cell migration	PTGS2 HIF1A KDR PPARG
6.93E-07	6	193	50.62028127	Reg. of blood pressure	NOS2 PTGS2 PPARG REN CYP11B1 CYP11B2
3.30E-05	5	259	31.43408715	Hormone-mediated signaling pathway	JAK2 NR3C1 PPARG REN NR3C2
3.30E-05	5	259	31.43408715	Hormone metabolic proc.	HIF1A REN CYP17A1 CYP11B1 CYP11B2
4.86E-05	5	290	28.07389163	Cellular ketone metabolic proc.	PTGS2 PPARG CYP17A1 CYP11B1 CYP11B2
2.23E-06	7	545	20.91376147	Blood circulation	NOS2 PTGS2 JAK2 PPARG REN CYP11B1 CYP11B2
2.27E-05	6	473	20.65478707	Response to peptide hormone	PTGS2 JAK2 PPARG CYP11B1 CYP11B2 PTPN1
3.12E-06	7	581	19.61790017	Reg. of hormone levels	NOS2 JAK2 HIF1A REN CYP17A1 CYP11B1 CYP11B2
9.02E-07	8	715	18.21858142	Cellular response to hormone stimulus	JAK2 NR3C1 PPARG REN NR3C2 CYP11B1 CYP11B2 PTPN1
4.63E-06	7	632	18.03481013	Circulatory system proc.	NOS2 PTGS2 JAK2 PPARG REN CYP11B1 CYP11B2
3.33E-08	10	998	16.31548812	Response to hormone	NOS2 PTGS2 JAK2 NR3C1 PPARG REN NR3C2 CYP11B1 CYP11B2 PTPN1
7.03E-05	7	1017	11.20747296	Response to lipid	NOS2 PTGS2 JAK2 NR3C1 PPARG REN NR3C2
2.06E-06	9	1313	11.16113589	Cellular response to oxygen-containing compound	NOS2 PTGS2 JAK2 HIF1A NR3C1 PPARG CYP11B1 CYP11B2 PTPN1
4.63E-06	9	1505	9.737256763	Cellular response to endogenous stimulus	PTGS2 JAK2 NR3C1 PPARG REN NR3C2 CYP11B1 CYP11B2 PTPN1
1.52E-06	10	1769	9.204554631	Response to endogenous stimulus	NOS2 PTGS2 JAK2 NR3C1 PPARG REN NR3C2 CYP11B1 CYP11B2 PTPN1
1.83E-06	10	1832	8.888022458	Response to oxygen-containing compound	NOS2 PTGS2 JAK2 HIF1A NR3C1 PPARG REN CYP11B1 CYP11B2 PTPN1

Table S4: Gene Ontology Enrichment (Molecular Function)

Enrichment FDR	nGenes	Pathway Genes	Fold Enrichment	Pathway	Genes
0.000218607	2	10	325.6571429	Oxidoreductase activity, acting on paired donors, with incorporation or reductio	CYP11B1 CYP11B2
6.86E-05	3	43	113.6013289	Steroid hydroxylase activity	CYP17A1 CYP11B1 CYP11B2
7.33E-05	3	47	103.9331307	Hsp90 protein binding	HIF1A NR3C1 KDR
0.001753405	2	32	101.7678571	Steroid hormone receptor activity	NR3C1 NR3C2
8.99E-05	3	59	82.79418886	Nuclear receptor activity	NR3C1 PPARG NR3C2
8.99E-05	3	59	82.79418886	Ligand-activated transcription factor activity	NR3C1 PPARG NR3C2
0.003717429	2	48	67.8452381	E-box binding	HIF1A PPARG
0.004107132	2	52	62.62637363	NADP binding	NOS2 HSD11B1
3.32E-08	6	159	61.44474394	Heme binding	NOS2 PTGS2 JAK2 CYP17A1 CYP11B1 CYP11B2
3.32E-08	6	169	57.80896027	Tetrapyrrole binding	NOS2 PTGS2 JAK2 CYP17A1 CYP11B1 CYP11B2
2.78E-05	4	121	53.82762692	Monooxygenase activity	NOS2 CYP17A1 CYP11B1 CYP11B2
0.000436937	3	106	46.08355795	Steroid binding	NR3C1 HSD11B1 NR3C2
3.12E-06	5	184	44.24689441	Oxidoreductase activity, acting on paired donors, with incorporation or reductio	NOS2 PTGS2 CYP17A1 CYP11B1 CYP11B2
0.001134331	3	150	32.56571429	Heat shock protein binding	HIF1A NR3C1 KDR
0.001499465	3	169	28.90448014	Iron ion binding	CYP17A1 CYP11B1 CYP11B2
8.99E-05	6	835	11.70025663	Oxidoreductase activity	NOS2 PTGS2 HSD11B1 CYP17A1 CYP11B1 CYP11B2
7.33E-05	7	1238	9.206785137	Transition metal ion binding	NR3C1 PPARG CYP17A1 NR3C2 CYP11B1 CYP11B2 PTPN1
0.006251959	4	712	9.147672552	Protein kinase binding	JAK2 HIF1A NR3C1 PTPN1
0.008903757	4	794	8.202950702	Kinase binding	JAK2 HIF1A NR3C1 PTPN1
0.009152318	4	811	8.03100229	Lipid binding	NR3C1 HSD11B1 PPARG NR3C2

Table S5: Gene Ontology Enrichment (Cellular Component)

Enrichment FDR	nGenes	Pathway Genes	Fold Enrichment	Pathway	Genes
0.000409213	2	6	542.7619048	Sorting endosome	KDR PTPN1
0.0254974	1	8	203.5357143	Mitochondrial crista	PTPN1
0.040167368	1	16	101.7678571	Cytoplasmic side of endoplasmic reticulum membrane	PTPN1
0.012048076	2	91	35.78649922	Caveola	PTGS2 JAK2
0.016898559	2	125	26.05257143	Plasma membrane raft	PTGS2 JAK2
0.033070286	2	206	15.80859917	RNA polymerase II transcription regulator complex	HIF1A PPARG
0.012048076	3	360	13.56904762	Membrane raft	PTGS2 JAK2 KDR
0.012048076	3	360	13.56904762	Membrane microdomain	PTGS2 JAK2 KDR
0.015348826	3	422	11.57549086	Receptor complex	KDR PPARG NR3C2
0.010675967	4	627	10.38778765	Organelle inner membrane	PTGS2 CYP11B1 CYP11B2 PTPN1
0.024515721	3	558	8.75422427	Mitochondrial inner membrane	CYP11B1 CYP11B2 PTPN1
0.010675967	5	1294	6.291675867	Endoplasmic reticulum membrane	PTGS2 HSD11B1 CYP17A1 NR3C2 PTPN1
0.010675967	5	1299	6.267458485	Endoplasmic reticulum subcompartment	PTGS2 HSD11B1 CYP17A1 NR3C2 PTPN1
0.010675967	5	1316	6.186495875	Chromatin	JAK2 HIF1A NR3C1 PPARG NR3C2
0.010675967	5	1316	6.186495875	Nuclear outer membrane-endoplasmic reticulum membrane network	PTGS2 HSD11B1 CYP17A1 NR3C2 PTPN1
0.015348826	5	1632	4.988620448	Organelle subcompartment	PTGS2 HSD11B1 CYP17A1 NR3C2 PTPN1
0.033070286	4	1352	4.817413356	Organelle envelope	PTGS2 CYP11B1 CYP11B2 PTPN1
0.033070286	4	1352	4.817413356	Envelope	PTGS2 CYP11B1 CYP11B2 PTPN1
0.024515721	5	1918	4.244748994	Chromosome	JAK2 HIF1A NR3C1 PPARG NR3C2

Table S6: KEGG Pathways

Enrichment FDR	nGenes	Pathway Genes	Fold Enrichment	Pathway	Genes
4.05E-06	4	61	106.7728337	Steroid hormone biosynthesis	HSD11B1 CYP17A1 CYP11B1 CYP11B2
0.000374521	3	76	64.27443609	Leishmaniasis	NOS2 PTGS2 JAK2
0.006511094	2	51	63.85434174	Ovarian steroidogenesis	PTGS2 CYP17A1
0.00735051	2	59	55.19612591	VEGF signalling pathway	PTGS2 KDR
0.00735051	2	65	50.1010989	Cortisol synthesis and secretion	CYP17A1 CYP11B1
0.00735051	2	68	47.8907563	Chemical carcinogenesis	PTGS2 HSD11B1
0.00735051	2	70	46.52244898	Prolactin signalling pathway	JAK2 CYP17A1
0.008498229	2	79	41.22242315	EGFR tyrosine kinase inhibitor resistance	JAK2 KDR
0.009725351	2	89	36.59069021	PD-L1 expression and PD-1 checkpoint pathway in cancer	JAK2 HIF1A
0.009725351	2	92	35.39751553	Small cell lung cancer	NOS2 PTGS2
0.01165185	2	108	30.15343915	Th17 cell differentiation	JAK2 HIF1A
0.01165185	2	109	29.8768021	HIF-1 signaling pathway	NOS2 HIF1A
0.01165185	2	112	29.07653061	Toxoplasmosis	NOS2 JAK2
0.003669465	3	194	25.17967599	Kaposi sarcoma-associated herpesvirus infection	PTGS2 JAK2 HIF1A
0.020741102	2	155	21.01013825	Cushing syndrome	CYP17A1 CYP11B1
0.025928479	2	179	18.19313647	Tuberculosis	NOS2 JAK2
0.031050721	2	202	16.12164074	Proteoglycans in cancer	HIF1A KDR
0.000374521	5	530	15.36118598	Pathways in cancer	NOS2 PTGS2 JAK2 HIF1A PPARG
0.032977389	2	214	15.2176235	Lipid and atherosclerosis	JAK2 PPARG
0.003669465	6	1527	6.397979231	Metabolic pathways	NOS2 PTGS2 HSD11B1 CYP17A1 CYP11B1 CYP11B2
